# Rab11fip5 regulates telencephalon development via ephrinB1 recycling

**DOI:** 10.1242/dev.196527

**Published:** 2021-02-02

**Authors:** Jaeho Yoon, Jerlin Garo, Moonsup Lee, Jian Sun, Yoo-Seok Hwang, Ira O. Daar

**Affiliations:** Cancer and Developmental Biology Laboratory (CDBL), Center for Cancer Research (CCR) – Frederick, National Cancer Institute, Frederick, MD 21702, USA

**Keywords:** Rab11fip5, EphrinB1, Rab11, Telencephalon, Autism-spectrum disorder, *Xenopus laevis*

## Abstract

Rab11 family-interacting protein 5 (Rab11fip5) is an adaptor protein that binds to the small GTPase Rab11, which has an important function in endosome recycling and trafficking of cellular proteins to the plasma membrane. Rab11fip5 is involved in many cellular processes, such as cytoskeleton rearrangement, iron uptake and exocytosis in neuroendocrine cells, and is also known as a candidate gene for autism-spectrum disorder. However, the role of Rab11fip5 during early embryonic development is not clearly understood. In this study, we identified Rab11fip5 as a protein that interacts with ephrinB1, a transmembrane ligand for Eph receptors. The PDZ binding motif in ephrinB1 and the Rab-binding domain in Rab11fip5 are necessary for their interaction in a complex. EphrinB1 and Rab11fip5 display overlapping expression in the telencephalon of developing amphibian embryos. The loss of Rab11fip5 function causes a reduction in telencephalon size and a decrease in the expression level of ephrinB1. Moreover, morpholino oligonucleotide-mediated knockdown of Rab11fip5 decreases cell proliferation in the telencephalon. The overexpression of ephrinB1 rescues these defects, suggesting that ephrinB1 recycling by the Rab11/Rab11fip5 complex is crucial for proper telencephalon development.

## Abstract (precis)

**Summary:** Rab11fip5, genetic mutations of which are associated with autism spectrum disorders in humans, plays a role in regulating telencephalon development via recycling of the crucial cargo ephrinB1.

## INTRODUCTION

Eph/ephrin signaling regulates multiple biological processes during embryogenesis. Loss-of-function studies have demonstrated the involvement of Eph/ephrin signaling in cell-cell contact during morphogenetic events and how abnormal regulation of the Eph/ephrin system causes severe embryonic defects (Janes et al., 2008; Klein, 2012). Precise regulation of Eph receptor and ephrin ligand levels are therefore vital for proper embryonic development, and this is achieved through epigenetic, transcriptional, post-transcriptional and post-translational regulation (Arvanitis and Davy, 2012). Post-translational regulation including protease-mediated shedding, ubiquitination, degradation, endocytosis and recycling is a particularly effective means to control Eph/ephrin signaling (Pitulescu and Adams, 2010; Atapattu et al., 2014).

Our previous studies have demonstrated that flotillin-1, a potential scaffold protein within caveolar membranes, prevents cleavage of ephrinB2 by ADAM10, which affects neural tube closure (Ji et al., 2014). We have also shown that Smurf2, an E3 ubiquitin ligase, induces the ubiquitination and degradation of ephrinB1, which is essential for the separation of the ectoderm and mesoderm in developing *Xenopus* embryos (Park et al., 2011; Rohani et al., 2011; Fagotto et al., 2013; Hwang et al., 2013; Rohani et al., 2014). Other studies have shown that endocytosis of the ligand or receptor can terminate Eph/ephrin signaling. (Zimmer et al., 2003; Irie et al., 2005; Essmann et al., 2008; Nakayama et al., 2013). The Eph receptor-ephrin ligand complex induces the association of endocytosis machinery and vesicle internalization, which is important for various developmental processes such as synaptogenesis, intestinal cell positioning and blood vessel morphogenesis (Pitulescu and Adams, 2010). Studies also show that contact between EphB4 and ephrinB2 activates the Rac-dependent trans-endocytosis of ephrinB ligands, which subsequently induces cell retraction in fibroblasts (Marston et al., 2003). In addition, Rac and its specific guanine nucleotide exchange factor Tiam2 are key components of EphB2 trans-endocytosis, leading to EphB2-stimulated contact repulsion (Gaitanos et al., 2016). HD-PTP (also known as Ptpn23) is required for EphB2 recycling (Lahaie et al., 2019). Several studies have demonstrated the molecular mechanisms employed for endocytosis of Eph/ephrin proteins in diverse model systems (Parker et al., 2004; Cowan et al., 2005; Zhuang et al., 2007; Deininger et al., 2008; Yoo et al., 2010; Xu and Wilkinson, 2013; Evergren et al., 2018). Recently, Rab5 was implicated in ephrinB1-dependent macro-pinocytosis and trans-endocytosis required for *Xenopus* endoderm ingression-type migration (Wen and Winklbauer, 2017). However, the specific role and identities of small GTPases and their adaptors, particularly regarding the ephrinB ligands, has been understudied.

Rab11 binds with various effector proteins that modulate trafficking processes like vesicle budding, transportation, docking and fusion to the plasma membrane (Meyers and Prekeris, 2002; Prekeris, 2003). One family of Rab11 effector proteins are the Rab11FIPs, which are known to link Rab11 with molecular motors, such as myosin V, and have a role in the directionality of vesicle transport (Nedvetsky et al., 2007; Laflamme et al., 2017). All Rab11FIPs have been classified and divided into three separate classes based on their highly conserved Rab11 binding domain (RBD) and other specific conserved domains. Class I Rab11FIPs have a distinctly conserved C2 domain with the ability for binding to the plasma membrane, whereas class II Rab11FIPs have a conserved calcium-binding EF-hand motif, and the class III Rab11FIPs have no specific conserved domains besides RBD. Rab11fip5, a Class I Rab11FIP, is known to regulate the trafficking of recycling endosomes to the apical membrane by interacting with Rab11 in polarized Madin-Darby Canine Kidney (MDCK) epithelial cells (Brown et al., 2000). Another study has shown that Rab11fip5 controls Rab11-mediated insulin granule exocytosis in pancreatic β-cells (Sugawara et al., 2009). However, the role of Rab11fip5 in developing vertebrate embryos is still unclear.

In this study, we show that Rab11fip5 displays a relatively restricted expression pattern that overlaps with ephrinB1 in the telencephalon of *Xenopus* tadpoles, and we provide evidence that the Rab11/Rab11fip5 complex regulates ephrinB1 recycling in the telencephalon. Genetic ablation or knockdown of Rab11fip5 causes a decrease in ephrinB1 levels, which in turn leads to reduced cell proliferation and defects in telencephalon development. The expression of wild-type Rab11fip5 rescues telencephalon size and ephrinB1 levels in Rab11fip5 morphants, whereas Rab11fip5-I603E, a Rab11 binding mutant, fails to rescue these defects. Furthermore, forced expression of ephrinB1 rescues telencephalon size in the Rab11fip5 morphants, indicating that ephrinB1 is a crucial cargo affecting telencephalon development. These results provide a novel mechanistic connection between the candidate autism spectrum disorder gene product, Rab11fip5, and ephrinB1, and indicate that proper recycling of ephrinB1 through the Rab11/Rab11fip5 complex controls proper telencephalon formation.

## RESULTS

### EphrinB1 complexes with Rab11fip5

**Fig. 1.**
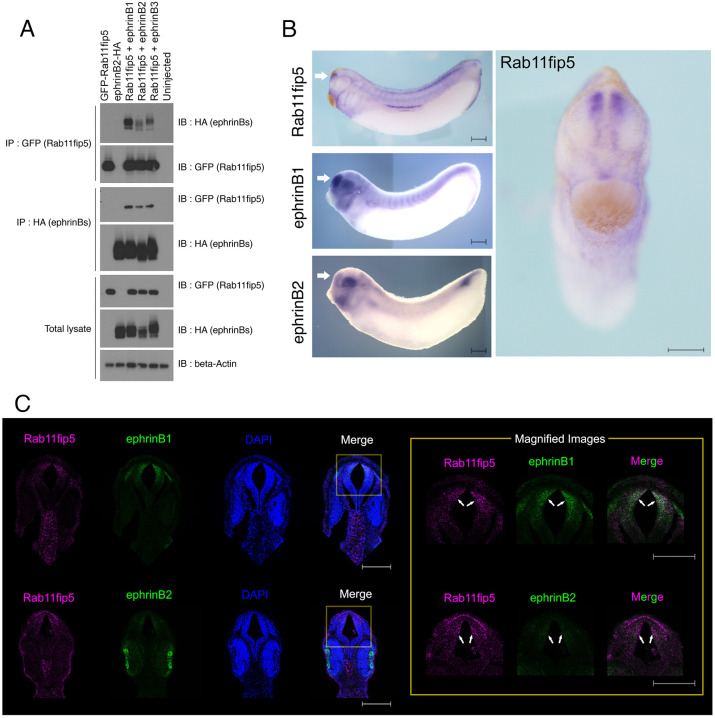
**EphrinB1 interacts with Rab11fip5.** (A) Co-IP was performed using stage-11 embryos injected with GFP-Rab11fip5 RNA (1 ng) and ephrinB1-HA (300 pg), ephrinB2-HA (500 pg) or ephrinB3-HA RNAs (300 pg). (B) Spatial expression pattern of Rab11fip5 and ephrinB1was analyzed using WISH at stage 28 with probes for Rab11fip5, ephrinB1 or ephrinB2. (C) HCR analysis was performed with anti-ephrinB1 or 2 and Rab11fip5 probe sets. The magnified images do not include DAPI for clarity. White arrows indicate the telencephalon. Scale bars: 200 µm.

To identify possible interaction partners involved in the regulation of ephrinB ligands, we performed immunoprecipitation (IP) and mass spectrometric analysis with HA-tagged ephrinB2 (ephrinB2-HA) overexpressed in *Xenopus* embryos. Several candidates were identified from the ephrinB2-HA immune complex, including metalloproteases, E3 ligases and genes involved in endocytosis and recycling (Fig. S1A,B). We focused on Rab11fip5 as it is known to play a role in the trafficking of cellular proteins from recycling endosomes to the plasma membrane along with Rab11, and thus may represent a new member of the ephrinB regulatory machinery (Meyers and Prekeris, 2002; Laflamme et al., 2017). Although ephrinB2 was used in the original screening, we examined whether other ephrinB ligands interact with Rab11fip5, as these ligands have a highly conserved intracellular domain (Fig. S2A,B). Co-IP analysis using *Xenopus* embryos expressing the three HA-tagged ephrinB ligands confirmed that all ephrinB ligands interact with Rab11fip5 (Fig. 1A).

To assess which ephrinB endogenously interacts with Rab11fip5 during embryonic development, we examined the spatial expression pattern of Rab11fip5 as well as ephrinB2. Consistent with the previous study (Wallkamm et al., 2016), Rab11fip5 transcripts were displayed in the telencephalon, using whole-mount *in situ* hybridization (WISH) (Fig. 1B) and hybridization chain reaction (HCR) (Fig. 1C). EphrinB1 also shows robust expression in the telencephalic region, whereas ephrinB2 displays strong expression in the eye and cranial neural crest cells, but is only weakly expressed in the telencephalon (Fig. 1B,C). EphrinB3 is known to be expressed in the midbrain and hindbrain, but not in the telencephalon (Helbling et al., 1999). These findings led us to speculate that ephrinB1 and Rab11fip5 are most likely to display an association in the developing telencephalon.

### The PDZ binding motif of ephrinB1 binds with the RBD domain of Rab11fip5

**Fig. 2.**
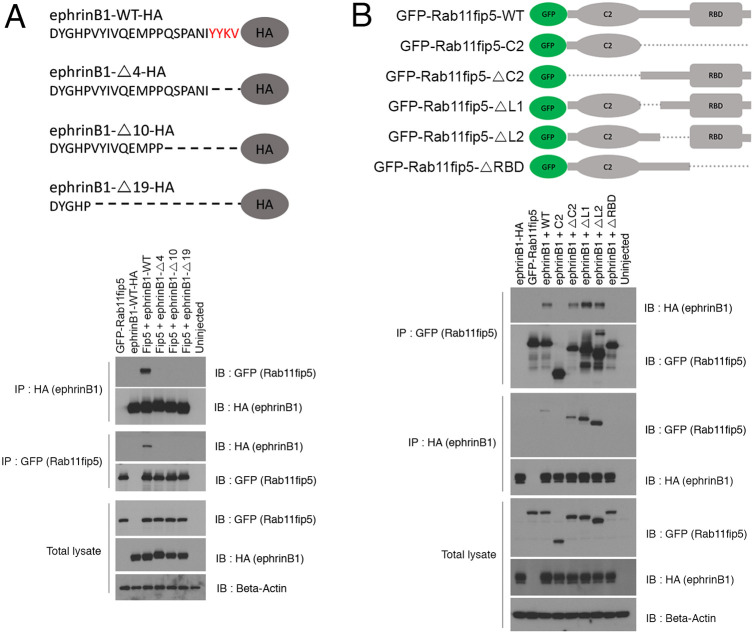
**Interaction domain mapping between ephrinB1 and Rab11fip5.** (A) Schematic of ephrinB1-WT or deletion mutants lacking 4, 10 or 19 amino acids from the C terminus. Co-IP was performed using stage-11 embryos injected with GFP-Rab11fip5 RNA (1 ng) and ephrinB1 deletion mutants (300 pg each) as indicated. The PDZ binding motif is in red. (B) Schematic of Rab11fip5-WT or deletion mutants lacking: the regions C-terminal to C2 (C2), C2 (ΔC2), the linker region 1 or 2 (ΔL1 or ΔL2), or the RBD domain (ΔRBD). Co-IP assay was performed using stage-11 embryos injected with ephrinB1-HA RNA (300 pg) and Rab11fip5 deletion mutants (1 ng each).

Various studies have demonstrated that ephrinB1 interacts with multiple downstream molecules such as Dsh, PDZ-RGS3, syntenin and GRIP through specific regions of the ephrinB1 intracellular domain (Bruckner et al., 1999; Lin et al., 1999; Tanaka et al., 2003; Lee et al., 2006; Qiu et al., 2008; McClelland et al., 2009; Yoon et al., 2018). The conserved domains of Rab11fip5 are also known to bind with several proteins, including the protein complex named factors for endosome recycling and Rab interactions (FERARI), 14-3-3, γ-SNAP and Rab11 (Tani et al., 2003; Li et al., 2014; Laflamme et al., 2017; Solinger et al., 2020). To determine the regions within ephrinB1 that are necessary for an interaction with Rab11fip5, we generated serial deletion mutants from the C terminus of ephrinB1 and co-expressed them in *Xenopus* embryos. Co-IP analysis with the deletion mutants indicated that the deletion of the last four amino acids, encoding the PDZ binding motif, is required for the interaction with Rab11fip5 (Fig. 2A). In a parallel experiment, we also generated serial deletion mutants of Rab11fip5 to identify the region necessary for the association with ephrinB1. Co-IP with the deletion mutants of Rab11fip5 showed that the RBD domain is required for binding with ephrinB1 (Fig. 2B).

### Rab11fip5 interacts with ephrinB1 via GTP-bound Rab11

**Fig. 3.**
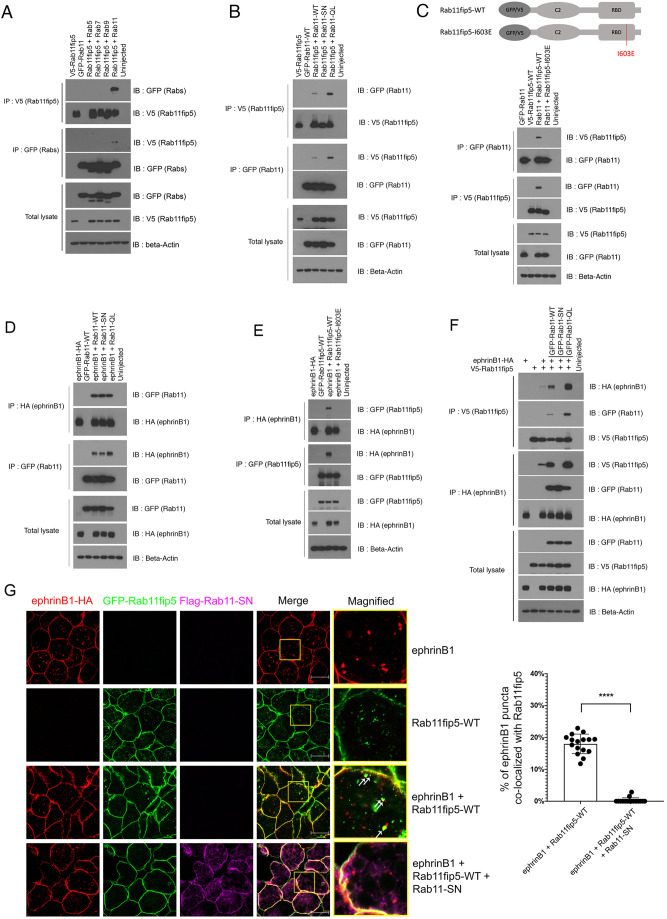
**Rab11fip5 interacts with ephrinB1 through GTP-bound Rab11.** (A) Co-IP assay was performed using stage-11 embryos injected with V5-Rab11fip5 RNA (1 ng) and GFP-Rab5, 7, 9 and 11 (1 ng each). (B) Co-IP assay was performed using stage-11 embryos injected with V5-Rab11fip5 RNA (1 ng) and GFP-Rab11-WT, dominant negative mutant (SN) or constitutively active mutant (QL) (1 ng each). (C) Co-IP assay was performed using stage-11 embryos injected with ephrinB1-HA RNA (300 pg) and GFP-Rab11-WT, SN or QL (1 ng each), as indicated. Schematic (top) shows Rab11fip5-WT or Rab11fip5-I603E mutant. (D) Co-IP assay using stage-11 embryos injected with GFP-Rab11 RNA (1 ng) and V5-Rab11fip5-WT or Rab11fip5-I603E mutant (1 ng each). (E) Co-IP assay was performed using stage-11 embryos injected with ephrinB1-HA RNA (300 pg) and GFP-Rab11fip5-WT or Rab11fip5-I603E mutant (1 ng each). (F) Co-IP assay was performed using stage-11 embryos injected with ephrinB1-HA RNAs (300 pg) and GFP-Rab11fip5 (1 ng) along with GFP-Rab11-WT, SN or QL (1 ng each). (G) Immunostaining was performed with ectodermal explants from stage-11 embryos injected with ephrinB1-HA (300 pg), GFP-Rab11fip5 (1 ng) and Flag-Rab11-SN (1 ng) as indicated. White arrows denote the colocalization of ephrinB1 and Rab11fip5-WT. Histogram depicts the percentage of ephrinB1 puncta colocalized with Rab11fip5 (*n*=16). Data are mean±s.d. of three individual experiments. ****P<0.0001 (two-tailed unpaired *t*-test). Scale bars: 20 µm.

Previous studies have demonstrated that Rab11fip5 acts as an effector protein for Rab11 and specifically binds to GTP-bound Rab11, an active form of Rab11 in mammalian cells (Tani et al., 2003). Crystal structure of Rab11 and Rab11FIP family proteins suggests that the RBD domain, which is highly conserved in all Rab11FIP family proteins, directly interacts with Rab11 and that conformational changes mediated by GTP/GDP cycling is crucial in the interaction (Jagoe et al., 2006). We confirmed the specific interaction between GTP-Rab11 and Rab11fip5 in developing embryos. Co-IP results showed that Rab11fip5 specifically binds with Rab11 but not related Rabs (Fig. 3A). Active Rab11 (GTP-Rab11) showed a robust interaction with Rab11fip5, but an inactive form of Rab11 (GDP-bound Rab11) did not interact with Rab11fip5 (Fig. 3B). Interestingly, previous reports have shown that a single amino acid substitution in the RBD domain, in which the highly conserved hydrophobic isoleucine (I) residue is replaced with a hydrophilic glutamic acid (E), abolishes the interaction between Rab11fip5 and Rab11 (Meyers and Prekeris, 2002; Junutula et al., 2004). Thus, we generated the same mutation in *Xenopus* Rab11fip5 (I603E) and confirmed using Co-IP that this mutation abolished the association between Rab11fip5 and Rab11 (Fig. 3C) (Meyers and Prekeris, 2002; Junutula et al., 2004). Consistent with the Co-IP results, wild-type Rab11fip5 colocalized with GFP-tagged Rab11 in recycling endosomes, whereas none of the Rab11fip5-I603E mutant showed colocalization with the Rab11 in recycling endosomes (Fig. S3). These results support previous studies that the RBD domain of Rab11fip5 interacts with GTP-Rab11.

Unlike Rab11fip5, ephrinB1 interacts with all forms of Rab11: wild type (WT), a dominant-negative form (S25N, SN) and a constitutively active form (Q70L, QL) (Fig. 3D). To determine the regions within Rab11 that are necessary for binding to ephrinB1, we generated a series of domain deletion mutants of Rab11 (Mulvaney et al., 2017). Co-IP analysis with the deletion mutants indicated that the hypervariable domain (HVD) is required for the interaction with ephrinB1 (Fig. S4). We then tested the interaction between ephrinB1 and the Rab11fip5-I603E mutant to verify whether the Rab11/Rab11fip5 interaction affects the ability of ephrinB1 to associate with Rab11fip5. Interestingly, our results demonstrated that the I603E mutation abolished the interaction between ephrinB1 and Rab11fip5 (Fig. 3E).

As our results suggested that Rab11 may mediate the interaction between ephrinB1 and Rab11fip5, we examined whether the GTP/GDP status of Rab11 affects the association between ephrinB1 and Rab11fip5. Interestingly, the overexpression of Rab11-WT noticeably increased the interaction between ephrinB1 and Rab11fip5, whereas the constitutively active Rab11-QL dramatically amplified the interaction. Conversely, the overexpression of the GDP-bound form, Rab11-SN, suppressed the interaction between ephrinB1 and Rab11fip5 (Fig. 3F). Consistent with the Co-IP result, ephrinB1 was detected in an array of endosomal puncta including endosomes and recycling endosomal vesicles in ectodermal explants (Fig. S5). About 20% of ephrinB1 endosomal puncta were colocalized with Rab11fip5 (Fig. 3G), and overexpression of Rab11-QL slightly elevated the colocalization (Fig. S5, white arrows). However, the overexpression of Rab11-SN suppresses endosome localization of Rab11fip5, suggesting an association with the recycling of ephrinB1 (Fig. 3D; Fig. S5). Our results indicate that the active form of Rab11 mediates the interaction of ephrinB1 and Rab11fip5.

### Knockout of Rab11fip5 causes a reduction in the telencephalon size, similar to an ephrinB1 knockdown

**Fig. 4.**
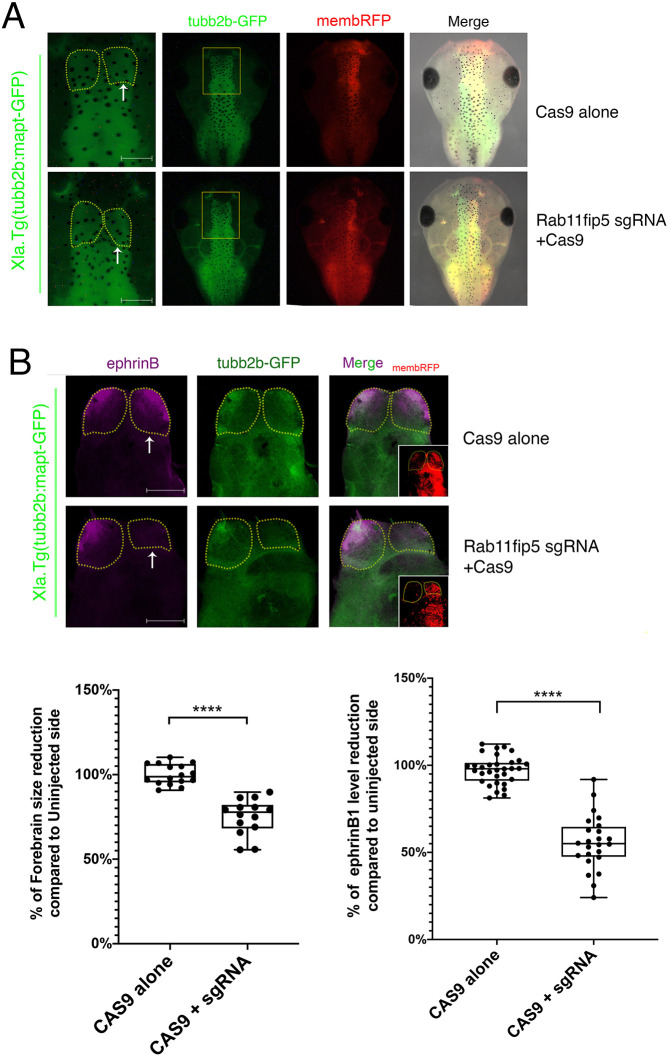
**Knockout of Rab11fip5 reduces developing telencephalon size and ephrinB1 expression levels.** (A) Cas9 protein alone or Cas9 with Rab11fip5 sgRNA along with membrane-RFP RNAs (100 pg) were injected into one blastomere of tubb2b: GFP transgenic embryos at the two-cell stage. Images were taken at stage 45 under fluorescent stereomicroscopy. Yellow dotted lines indicate telencephalon borders. White arrows indicate injected side. Left panels shows magnification of boxed areas in right panels. (B) Brains were dissected from Rab11fip5 KO tadpoles at stage 45. Immunostaining was performed with anti-ephrinB1 antibodies. Insets show red fluorescent protein (RFP), indicating injected side. Histograms depict relative telencephalon size (*n*=15; left) and endogenous ephrinB1 levels (*n*=24; right) compared with uninjected telencephalon. Data are mean±s.d. from three individual experiments. Box plots shows the median values (middle bars) and first to third interquartile ranges (boxes); whiskers indicate minimum/maximum; dots indicate individual data points; asterisks indicate significant difference. ****P<0.0001 (two-tailed unpaired *t*-test). Scale bars: 200 µm.

As the role of Rab11fip5 in developing embryos and its relationship to ephrinB1 is unknown, Rab11fip5 loss-of-function studies were performed. We employed a knockout strategy using CRISPR/Cas9 to explore the developmental role of Rab11fip5. The single-guide RNA (sgRNA) targeted the first exon of the Rab11fip5 gene (Fig. S6A). Cas9 proteins and sgRNA were injected into one blastomere of two-cell-stage transgenic embryos (tubb2b: mapt-GFP), which have a neural-β-tubulin promoter and 5′ untranslated region (UTR) driving expression of GFP in neural tissues, including the brain. DNA sequencing results of the targeted genomic region in Rab11fip5 knockout tadpoles showed perturbation of sequencing peaks, which indicated several nucleotide deletions near the PAM region (Fig. S6B). At stage 45, the tadpoles expressing only Cas9 showed a similar telencephalon size to the uninjected control side. Interestingly, the knockout of Rab11fip5 caused a significant reduction in the telencephalon size (∼25%); however, the size of the mesencephalon and rhombencephalon were not affected (Fig. 4A).

To determine whether this phenotype may also exist when ephrinB1 expression is compromised, we performed ephrinB1 loss-of-function analysis using morpholino oligos (MO) in the tubb2b: mapt-GFP transgenic tadpoles (Fig. S7). Dissected brains from stage 45 tadpoles were immunostained using antibodies that recognize a conserved epitope residing in the C-terminal 19 amino acids (CPHYEKVSGDYGHPVYIVQ) of the intracellular domain in all three ephrinBs. We observed that ephrinB ligands are expressed in the telencephalon and ephrinB1 MO significantly reduced ephrinB expression levels in the telencephalon, which suggests that ephrinB1 is the major ephrinB ligand expressed in the telencephalon. The ephrinB1 morphants, like the Rab11fip5 knockout tadpoles, displayed a noteworthy reduction in the telencephalon size (Fig. 4B; Fig. S7). Furthermore, immunostaining of dissected brains from Rab11fip5 knockout tadpoles showed that the amount of ephrinB1 was reduced by ∼40% (Fig. 4B). Our results suggest that Rab11fip5 is required to maintain ephrinB1 expression levels as well as proper telencephalon size.

### Rab11fip5 regulates ephrinB1 recycling

**Fig. 5.**
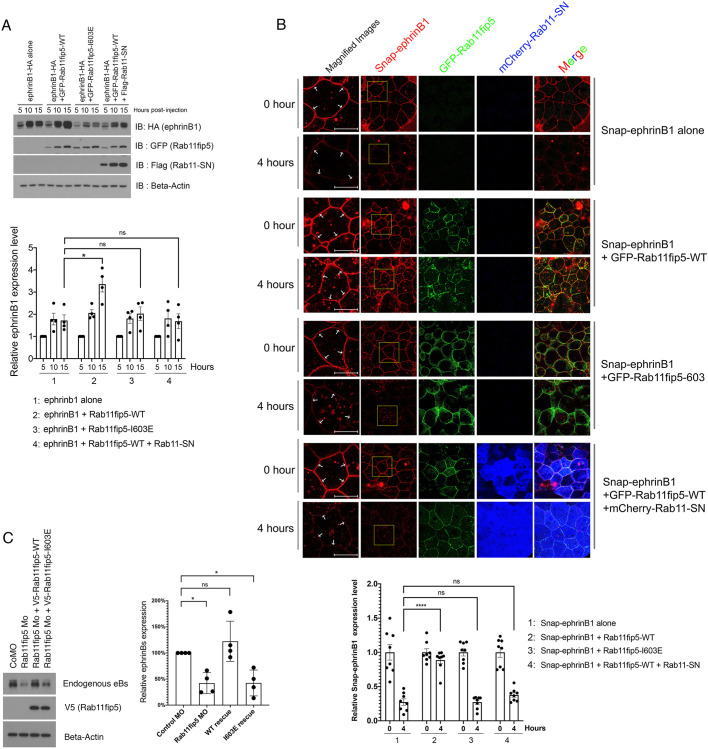
**Rab11fip5 regulates ephrinB1 recycling.** (A) RNAs were injected into two D1 blastomeres at the eight-cell stage as indicated. Embryos were harvested at 5, 10 and 15 h after injection and western blot analysis was performed. Histogram depicts relative ephrinB1 levels at 15 h post-injection (*n*=4). (B) RNAs were injected into two D1 blastomeres at the eight-cell stage as indicated. Ectodermal explants were dissected at stage 11 and then membrane-ephrinB1 was stained using Snap-tag substrate. Live cell images were taken at 0 h and 4 h post-Snap-staining. White arrows denote the fluorescent labeled membrane Snap-ephrinB1. Histogram shows relative membrane ephrinB1 levels (*n*=8). Left panels shows magnification of boxed areas in right panels. (C) MOs and RNAs as indicated were injected into two D1 blastomeres in eight-cell-stage embryos. Five brains were harvested in each group at stage 45 and western blot analysis performed using anti-ephrinB1 and anti-V5 antibodies. β-Actin was used as a loading control. Histogram shows relative membrane-bound ephrinB1 levels (*n*=4). Data are mean±s.d. of three individual experiments. *P<0.05, ****P<0.0001 [one-way ANOVA (Dunnett's multiple comparisons test)]. ns, no statistical differences between the groups. Scale bars: 10 µm.

The Rab11/Rab11fip5 complex plays a key role in vesicular trafficking from endosomes to the plasma membrane (Hales et al., 2001). This trafficking is crucial for regulating the expression level of membrane proteins, such as receptors and ion channels (Das et al., 2018). Thus, to determine whether Rab11fip5 might affect ephrinB1 recycling, Rab11fip5-WT, the I603E mutant and/or dominant negative Rab11 were co-expressed along with ephrinB1-HA. Injected embryos were harvested at 5, 10 and 15 h after injection. Five hours post-injection, ephrinB1 protein was detected in developing embryos. Co-expression of Rab11fip5-WT markedly elevates exogenously expressed ephrinB1 levels 15 h after RNA injection, whereas the Rab11fip5-I603E mutant failed to enhance the ephrinB1 levels (Fig. 5A). Moreover, overexpression of Rab11-QL increased ephrinB1 levels slightly above those observed for Rab11-WT overexpression (Fig. S8). However, co-expression of Rab11-SN along with Rab11fip5-WT inhibited the enhancement of ephrinB1 levels observed at 10 h and 15 h post-injection (Fig. 5A; Fig. S8). This result strongly suggests that the increase in exogenously expressed ephrinB1 induced by Rab11fip5 is dependent upon Rab11, which is a key molecule for the process of vesicle recycling.

To further test the role of Rab11fip5 in the recycling of ephrinB1 to the plasma membrane, neuroectoderm tissue was dissected from embryos expressing Snap-tagged-ephrinB1 (Snap-ephrinB1). Membrane-bound Snap-ephrinB1 proteins were fluorescently labeled using a non-cell permeable Snap-tag substrate (Snap^TM^-Surface 647). Live cell imaging showed that Snap-ephrinB1 levels were dramatically reduced after 4 h of culture. Strikingly, co-expression of GFP-tagged Rab11fip5-WT maintained the levels of fluorescently labeled ephrinB1 after 4 h post-fluorescent labeling (Fig. 5B; Fig. S9). In contrast, co-expression of the GFP-tagged Rab11fip5-I603E mutant or co-expression of both the Rab11fip5-WT along with Rab11-SN did not sustain the Snap-ephrinB1 levels (Fig. 5B). These results indicate that the Rab11fip5/Rab11 complex regulates ephrinB1 recycling to the plasma membrane.

Next, we designed specific MOs against Rab11fip5 to further validate the loss-of-function phenotype previously observed by the genetic ablation of Rab11fip5. We confirmed the ability of the Rab11fip5 MO to block translation of exogenously expressed protein (Fig. S10). The expression level of endogenous ephrinB ligands was analyzed to assess whether knockdown of Rab11fip5 showed similar effects to those found with CRISPR/Cas9-mediated genetic ablation. MOs and RNAs were targeted to neural tissue by injection into two D1 blastomeres at the eight-cell stage, and brains were dissected from stage 45 tadpoles for western blot analysis. The brains from Rab11fip5 morphants showed decreased levels of the ephrinB1 ligand, which is rescued by the co-injection of MO-resistant Rab11fip5-WT RNA (Fig. 5C). However, expression of the Rab11fip5-I603E mutant that cannot bind Rab11 nor ephrinB1 failed to rescue the levels of endogenous ephrinB1. This Rab11fip5 loss-of-function data further supports the concept that the Rab11fip5/Rab11 complex regulates ephrinB1 recycling.

### MO-mediated knockdown of Rab11fip5 causes a decrease in cell proliferation and telencephalon size

**Fig. 6.**
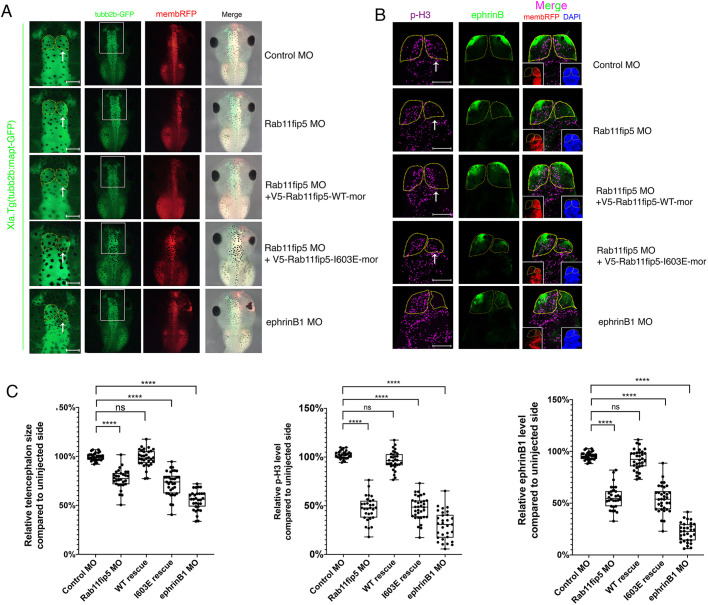
**MO-mediated knockdown of Rab11fip5 reduces ephrinB1 levels, causes reduction in telencephalon size and suppresses proliferation.** (A) MOs (4 ng) and RNAs (200 pg) as indicated were injected into one D1 blastomere of tubb2b: GFP transgenic embryos at the eight-cell stage. Images were taken at stage 45 using fluorescent stereomicroscopy. Yellow dotted lines indicate telencephalon borders. White arrows indicate injected side. Left panels shows magnification of boxed areas in right panels. (B) MOs (4 ng) and RNAs (200 pg) were injected into one D1 blastomere at the eight-cell stage as indicated. Brains were dissected at stage 45 and immunostaining performed using anti-ephrinB1 and phospho-histone H3 antibodies. Insets show red fluorescent protein (RFP), indicating injected side, and DAPI. (C) Histograms show relative telencephalon size (left), ephrinB1 expression levels (right) and phospho-histone H3 levels (middle) compared with uninjected telencephalons (*n*=32). Data are mean±s.d. from three individual experiments. Box plots shows the median values (middle bars) and first to third interquartile ranges (boxes); whiskers indicate minimum/maximum; dots indicate individual data points; asterisks indicate significant difference. ****P<0.0001 [one-way ANOVA (Dunnett's multiple comparisons test)]. ns, no statistical differences between the groups. Scale bars: 200 µm.

To assess whether the MO-mediated Rab11fip5 knockdown also leads to a reduced telencephalon size, similar to the genetic ablation of Rab11fip5 or ephrinB1 morphants, MOs were injected into one D1 blastomere of tubb2b: GFP transgenic embryos at the eight-cell stage (Fig. 6A). Rab11fip5 morphants show reduced telencephalon size that is consistent with the Rab11fip5 knockout tadpoles (Fig. 6A). This defect is rescued by the reintroduction of an MO-resistant Rab11fip5-WT RNA. However, the Rab11fip5-I603E interaction mutant failed to rescue the telencephalon size (Fig. 6A). Although these data strongly support the concept that reduced ephrinB1 expression in Rab11fip5 morphants is due to ephrinB1 protein regulation, we tested whether Rab11fip5 knockdown affects ephrinB1 expression at the transcriptional level. Unlike ephrinB1 protein levels, RT-qPCR analysis showed that ephrinB1 mRNA level was actually slightly increased in Rab11fip5 knockdown embryos (Fig. S11). These results confirmed that Rab11fip5 regulates ephrinB1 protein levels, which in turn may affect the telencephalon size.

Next, we questioned how loss of Rab11fip5 decreases telencephalon size. One possibility may be defects in early forebrain specification. However, our WISH results showed that forebrain markers including anti-BF-1, OTX2 or PAX6 were unchanged by knockdown of Rab11fip5 or ephrinB1 (Fig. S12). An alternative possibility may be through suppression of proliferation in this tissue. This concept is supported by knockout mouse studies. The cortices from EphA4^−^/^−^ mice showed reduced cerebral wall thickness (North et al., 2009). Interestingly, knockout of ephrinB1 reduced EphA4 activation, which is vital for cell proliferation in cortical proliferative zones resulting in smaller cortical area (North et al., 2009). The proliferation of neuronal progenitor cells in the developing cerebral cortex is achieved through activating EphA4 receptors or PDZ-RGS3 (Qiu et al., 2008). Thus, we examined phospho-histone H3, a well-known proliferation marker, in the tadpole brain to evaluate whether Rab11fip5 affects cell proliferation. Knockdown of Rab11fip5 causes a reduction in the size of the telencephalon, as well as a decrease in ephrinB1 protein levels, and suppression of cell proliferation compared with the uninjected side of telencephalons (Fig. 6B,C). All these deficits are rescued by the introduction of an MO-resistant Rab11fip5-WT RNA. In contrast, the Rab11fip5-I603E interaction mutant failed to rescue these defects (Fig. 6B,C). In addition, we also tested whether Rab11fip5 knockdown reduces telencephalon size by inducing apoptosis. However, elevated apoptosis was not observed in developing telencephalon of early-and late-stage Rab11fip5 or ephrinB1 morphant embryos (Fig. S13A,B). Together, these data strongly suggest that Rab11fip5 is required for cell proliferation in the telencephalon.

### Rab11fip5 regulates cell proliferation in the developing telencephalon through control of ephrinB1 levels

**Fig. 7.**
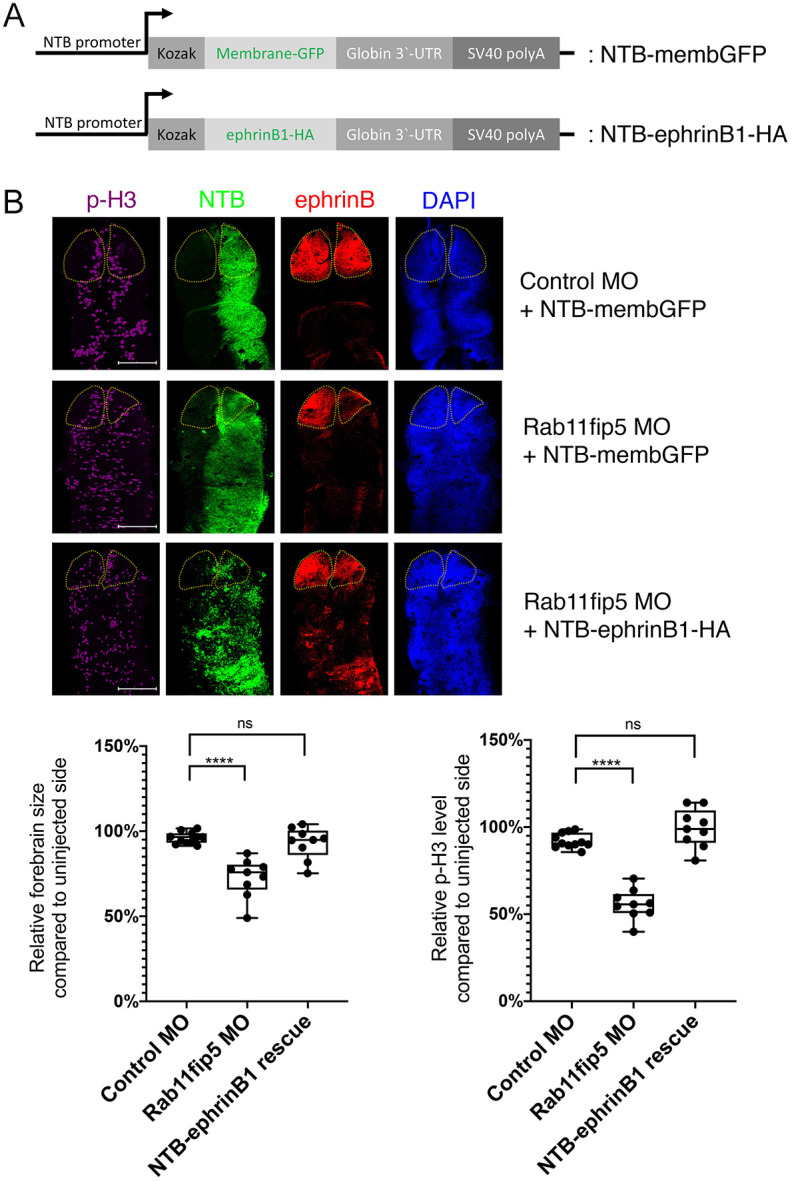
**Overexpression of ephrinB1 rescues Rab11fip5 knockdown defects.** (A) Schematic of NTB-membrane GFP and NTB-ephrinB1-HA clones. (B) MOs (4 ng) and NTB constructs (50 pg) were injected into one D1.1 blastomere at the 16-cell stage as indicated. Brains were dissected at stage 35 and immunostaining performed using anti-ephrinBs and phospho-histone H3 antibodies. In the NTB panels, overexpressed ephrinB1-HA was stained with anti-HA-Alexa Fluor 488, whereas membrane GFP was stained with anti-GFP-Alexa Fluor 488. Yellow dotted lines indicate telencephalon borders. Histograms show relative telencephalon size (left) and phospho-histone H3 levels (right) compared with uninjected telencephalons (*n*=9). Data are mean±s.d. from three individual experiments. Box plots shows the median values (middle bars) and first to third interquartile ranges (boxes); whiskers indicate minimum/maximum; dots indicate individual data points; asterisks indicate significant difference. ****P<0.0001 [one-way ANOVA (Dunnett's multiple comparisons test)]. ns, no statistical differences between the groups. Scale bars: 200 µm.

Our biochemical data and loss-of-function analyses have suggested that ephrinB1 may be a cargo of the Rab11-Rab11fip5 complex, and this regulation of ephrinB1 affects cell proliferation and thus brain size. One crucial test of this concept is to determine whether the telencephalon phenotype is rescued by overexpression of ephrinB1 in Rab11fip5 morphant tadpoles. Our previous study showed that the half-life of the ephrinB1 protein is relatively short, ∼2.5 h (Hwang et al., 2013). Consistent with this report, embryos injected with ephrinB1 RNA at the eight-cell stage did not display exogenous ephrinB1 expression in embryos after stage 30 and the later tailbud stages. This is most likely owing to a combination of *ephrinB1* mRNA degradation *in vivo* over time and the relatively short half-life of ephrinB1 protein. As using mRNA to overexpress ephrinB1 at high levels in early embryogenesis leads to multiple defects, we used another method to circumvent this issue. We expressed ephrinB1 in the appropriate tissue and stage of development by generating DNA constructs containing a neural β-tubulin (tubb2b) promoter with membrane GFP (NTB-membrane GFP) or HA-tagged ephrinB1 (NTB-ephrinB1-HA; Fig. 7A). MOs and NTB constructs were injected into the D1.1 blastomere, and the *Xenopus* brains were dissected at stage 45 (Fig. 7B). Immunostaining with anti-ephrinB antibodies showed that embryonic brains harboring the Rab11fip5 MO and the control NTB-membrane GFP construct have decreased levels of ephrinB1 and a reduced telencephalon size compared with the uninjected side (Fig. 7B). Interestingly, overexpressing ephrinB1 via the NTB-ephrinB1-HA construct in Rab11fip5 morphants rescued the telencephalon size, although these brains displayed a relatively low abundance of ephrinB1-HA owing to the effect of Rab11fip5 loss on ephrinB1 recycling (Fig. 7B). Furthermore, immunostaining using anti-phospho-histone H3-specific antibodies showed that Rab11fip5 morphant brains have reduced phospho-histone H3 levels compared with uninjected telencephalons, and this is also rescued by the NBT-ephrinB1-HA construct (Fig. 7B). Collectively, these results suggest that Rab11fip5 regulates ephrinB1 levels, which play an important role in cell proliferation in the developing telencephalon.

## DISCUSSION

The three ephrinB ligands show distinctive ectodomains that possess different binding affinities with the various cognate Eph receptors (Arvanitis and Davy, 2012). By contrast, the intracellular domain of the ephrinB ligands, particularly ephrinB1 and ephrinB2 (Fig. S2), has exceedingly conserved protein sequences. This conservation suggests that the ephrinBs may share intracellular signal transduction and protein homeostasis mechanisms, including endocytosis, ubiquitination and recycling. Endocytosis and recycling play crucial roles in Eph/ephrin signaling (Atapattu et al., 2014). When an Eph-receptor-bearing cell comes in contact with an ephrin-ligand-bearing cell, the Eph/ephrin complex undergoes endocytosis or trans-endocytosis (Pitulescu and Adams, 2010). This event is followed by degradation or recycling of the receptor/ligand to the plasma membrane, which terminates or re-initiates the signaling (Pitulescu and Adams, 2010). A few studies have demonstrated trafficking mechanisms involving Eph receptors (Boissier et al., 2013; Gundry et al., 2017), although the molecular mechanism for ephrin ligand recycling has been elusive. During the development of vertebrate embryos, the three ephrinB ligands show different spatial and temporal expression patterns (Helbling et al., 1999). Our transcript analysis indicates that the telencephalon of developing embryos predominantly expresses ephrinB1 (Fig. 1B), which has a reported function in neural cell proliferation (Qiu et al., 2008; North et al., 2009). Our results showed that the phospho-histone H3-positive cells are found adjacent to ephrinB1-expressing cells in the developing telencephalon. As indicated in knockout mice studies, ephrinB1 ligand appears to promote the proliferation of progenitor cells through Eph receptors (North et al., 2013). Consistent with a possible interaction between ephrinB1 and Rab11fip5, we found that Rab11fip5 is also expressed in the developing telencephalon (Fig. 1B). Rab11fip5 is an effector protein of Rab11, which associates with recycling machinery and controls the recycling of certain endocytosed receptor proteins (Prekeris, 2003).

Recycling is essential for controlling various cellular processes including cell growth, differentiation and cell-cell communication during embryogenesis (Maxfield and McGraw, 2004). Rab-GTPases, such as Rab11, Rab25 and Rab35 are known as key molecules for controlling recycling processes (Maxfield and McGraw, 2004). Rab11 forms a complex with diverse proteins such as EHD1 and Rab11FIP family members (Prekeris, 2003). There are limited studies examining the role of Rab11fip5 during embryonic development. One study demonstrated that a conditional knockout of Rab11fip5 in mice causes the failure of AMPA receptor recycling, resulting in severe long-term neuronal depression (Bacaj et al., 2015). Another study suggested that Rab11fip5 is involved in aPKC recycling to regulate cell polarity in *Drosophila* ectoderm (Calero-Cuenca and Sotillos, 2018).

The misregulation of trafficking processes leads to human diseases, such as developmental disorders and cancer (Bhuin and Roy, 2015; D'Agostino et al., 2019). Interestingly, one such developmental condition in which Rab11fip5 has been reported to play a role is autism spectrum disorders in humans. In the case of Rab11fip5, this disorder occurs when a chromosomal translocation causes total loss of Rab11fip5, or through a missense mutation in Rab11fip5 (Roohi et al., 2008; Rafi et al., 2019). It is interesting to consider that, like Rab11fip5, the ablation or reduction of other autism-related gene products leads to reduced telencephalon development in the *Xenopus* model system, even though the cellular processes regulated by these genes varies considerably. For example, the functions for some of these proteins ranges from roles in multiple microtubule-dependent cellular processes (Willsey et al., 2018, 2020), apoptosis and cell cycle pathways (Singh et al., 2020), to forming the polycomb repressive deubiquitination complex (Lichtig et al., 2020).

Although we have shown that loss of ephrinB1 affects cell proliferation rather than apoptosis or early brain specification, it is interesting to consider how this is accomplished. Mouse studies (Qiu et al., 2008; North et al., 2009) demonstrated that knockout of EphA4 results in suppressed proliferation and decreasing cell number in cultured cortical progenitor cells. *In vivo*, EphA4^−^/^−^ mice showed reduced cerebral wall thickness caused by less cell division, and ephrinB1 is crucial for initiating EphA4 signaling to promote cortical cell proliferation. Similar to results from the knockout study, our results also showed that phospho-histone H3-positive cells are found adjacent to ephrinB1-expressing cells. Therefore, we postulate that ephrinB1 activates Eph forward signaling resulting in enhanced cell proliferation and increasing telencephalon size. Future studies will still be required to assess the signaling mechanisms that lead to an increase in cell proliferation, and whether this process occurs via forward signaling through an Eph receptor or reverse signaling through the ligand.

We provide evidence that Rab11fip5 and ephrinB1 are co-expressed in the telencephalon of developing embryos and that Rab11fip5 interacts with ephrinB1 through Rab11. The loss of Rab11fip5 function decreases the ephrinB1 protein levels, which leads to a corresponding reduction in the size of the telencephalon. Moreover, our data indicate that ephrinB1 is necessary to maintain the normal proliferation of telencephalic cells in the developing *Xenopus* brain. Overall, our results indicate that proper recycling of ephrinB1 through the Rab11/Rab11fip5 complex controls proper telencephalon formation.

## MATERIALS AND METHODS

### Plasmids and reagents

The cDNA clone that encodes full-length Rab11fip5 was obtained from Source BioScience (GenBank ID: BC070758). V5-tagged deletion and point mutants of Rab11fip5 (C2, ΔC2, ΔL1, ΔL2, ΔRBD and I603E) in pCS107 vector were generated using QuikChange II Site-Directed Mutagenesis Kit (Agilent Technologies, 200521). Various HA-tagged mutants of ephrinB1 (Δ4, Δ10 and Δ19) have been reported (Lee et al., 2006). The sequences of MOs are as follows: ephrinB1 MO, 5′-GGAGCCCTTCCATCCGCACAGGTGG-3′; Rab11fip5 MO, 5′-CGAAGAAACATGAGGACGAGCC TCT-3′. The *Xenopus* transgenic frogs [Xla.Tg (tubb2b: mapt-GFP)] were obtained from the National *Xenopus* Resource (NXR). 3.8NBetaT-CAT was a gift from Paul Krieg (Addgene plasmid #17146). NBT-membrane GFP or ephrinB1-HA were constructed using ClaI and Asp718 restriction sites.

### Embryo injections

We obtained *Xenopus laevis* embryos using standard methods (Moody, 2000). mRNAs were produced using the SP6 mMessage mMachine Kit (Ambion). The doses are indicated in the figure legends. For the rescue of MO effects, MO-resistant mRNA constructs were designed and mRNA synthesized. In the case of Rab11fip5-Morpholino Resistant (MoR), the 5′ UTR is deleted, and four nucleotide substitutions were generated in wobble codons subsequent to the ATG start codon (Fig. S6). MOs and mRNAs were microinjected into the animal pole region in one-cell-stage embryos or the D1.1 blastomere at the eight-cell stage. Animal care and use for this study was performed in accordance with the recommendations of AAALAC for the care and use of laboratory animals in an AAALAC-approved facility. Experimental procedures were specifically approved by the Animal Care and Use Committee of the National Cancer Institute-Frederick (ASP #18-433) in compliance with AAALAC guidelines.

### IP and western blot analysis

*Xenopus* embryo lysates were prepared with ice-cold TNSG buffer [20 mM Tris-HCl (pH 7.5), 137 mM NaCl, 5 mM MgCl_2_ and 1% NP-40]. IPs were performed for 8 h with 25 embryo-equivalent extracts using GFP-Trap (Chromotek), monoclonal anti-HA or anti-V5-agarose (20 μl per sample, A2095 and A7345, Milipore Sigma). Western blot analysis was performed using anti-Flag-horseradish peroxidase (HRP)-conjugated (1:5000, A8592, Sigma-Aldrich), anti-HA-HRP-conjugated (1:5000, 12013819001, Roche), anti-GFP-HRP-conjugated (1:5000, 600-403-215, Rockland), and anti-beta-actin (1:2000, Santa Cruz Biotechnology) antibodies.

### Immunofluorescence microscopy

Brains were dissected at stage 45 and immunofluorescence microscopy was carried out using standard protocols. The following primary antibodies were used: anti-V5 (1:500, G189, ABM), anti-HA (1:1000, C29F4, Cell Signaling Technology), anti-Phospho-Histone H3 (1:1000, 9706, Cell Signaling Technology), anti-Cleaved Caspase-3 (1:1000, 9661, Cell Signaling Technology), and anti-ephrinBs (1: 3000, 600-401-MP0, Rockland).

### Membrane-bound ephrinB1 analysis using Snap-tag technique

MOs and RNAs were injected into the D1 blastomere at stage 8. Neuroectoderms were dissected from stage-10 embryos and incubated with Snap-tag substrate (Non-cell permeable, Snap-Surface^TM^ 647) for 30 min at room temperature in 1× Modified Barth's Saline (MBS) medium. After briefly washing with 1× MBS to remove excess Snap-tag substrate, explants were incubated in 1× MBS containing 0.5% bovine serum albumin and live cell images were taken using confocal microscopy (Zeiss LSM880).

### WISH

*Xenopus* embryos were collected at stage 28 for hybridization with the Rab11fip5, ephrinB1, ephrinB2, BF-1, OTX2 and Pax6 probes. Embryos were injected with dextran alexa-594 and various MOs to distinguish the injected side of embryos. The embryos were then processed for WISH using standard methods.

### HCR

HCR probe sets were designed by Molecular Instruments. *Xenopus* embryos were collected at stage 28, then processed for HCR using standard methods (Choi et al, 2010). Briefly, embryos were fixed with MEMFA (MOPS 0.1 M, EGTA 2 mM, MgSO_4_ 1 mM, formaldehyde 3.7%) and dehydrated with 100% MeOH, embryos were rehydrated with MeOH/PBST (1× PBS with 0.1% Triton X-100), and then washed with PBST. After pre-hybridization, probe mixture (5 pmol) was incubated at 37°C for 16 h, and then washed with the probe washing buffer and 5× saline sodium citrate with Triton X-100 (SSCT). For signal amplification, 50 pmol of each hairpin was incubated with embryos in amplification buffer at room temperature for 16 h. Embryos were washed with 5× SSCT and images were taken using confocal microscopy (Zeiss LSM880).

### Rab11fip5 knockout using CRISPR/CAS9

For guide RNA design, the design tool CRISPRdirect (https://crispr.dbcls.jp) application was used to scan the genome sequence for suitable CAS9 target sequences including a PAM site. As shown in Fig. S4, the sequences in the first exon of the Rab11fip5 (5′-CTGGGGCCTTGGAGCGGGCA-3′) on the reverse strand was selected, as no off-target effects were predicted. sgRNA template construction, *in vitro* transcription of sgRNA, microinjection and genotyping were performed as previously described (Nakayama et al., 2014). Small sections of the tail were prepared from F0 embryos and then analyzed to verify mutagenesis by the direct sequencing of PCR amplicons assay. Anterior parts, including brains of F0 embryos, were used for immunostaining.

### RNA isolation and RT-qPCR

For the RT-qPCR, total RNA was prepared using RNeasy™ Mini Kit (Qiagen, 74104), and cDNA was synthesized using the Superscript™ IV system (Invitrogen). The PCR reactions were performed with SYBR Green PCR Master Mix (Applied Biosystems™, 4364346) using QuantStudio5™ (Applied Biosystems™). ODC is used for normalization. Primer sequences: ephrinB1 Forward 5′-GCCCTAGCAAAGAGGCTGAT-3′, Reverse 5′-CTCCAATCGCTGCAAAGACG-3′; ODC Forward 5′-ATGCCAACCCATGCAAACAG-3′, Reverse 5′-TCACACTTAAACGAGCAGAGGA-3′.

### Statistical analysis

For all experiments, lineage tracer was co-injected with MOs and RNAs. Dead embryos and embryos which had mistargeted injection were excluded from all experimental analysis. Sample size was determined as indicated in the figures and a specific statistical method was not used. All experiments were performed blinded with order of testing randomized. ImageJ was used for all analysis including telencephalon size, ephrinB1 expression level and phospho-histone H3 levels. At least three independent experiments for each analysis were performed. Datasets were compared using Student's *t*-test (two-tailed, unequal variances) or a one-way ANOVA in Prism8. Cross comparisons were performed only if the overall *P*-value of the ANOVA was <0.05. Error bars indicate s.d.

## Supplementary Material

Supplementary information

Reviewer comments
