# Supplementary material for: Rab11fip5 regulates telencephalon development via ephrinB1 recycling
Source: Development. 2021 Feb 2;148(3):dev196527. doi: 10.1242/dev.196527 (PMC7875491; doi:10.1242/dev.196527)
Supplement: Supplementary information [file develop-148-196527-s1.pdf]

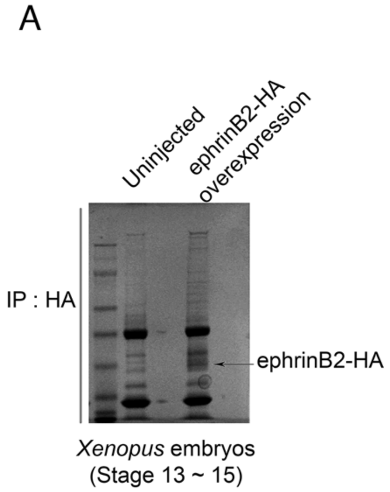

B Novel ephrinB2 binding proteins

Gene name	Protein	UniProt accession	# of peptides	
			ephrinB2-HA	Control
**Endocytosis / recycling related factors**				
rab11fip5	RAB11 Family Interacting Protein 5	Q6NRJ2	4	0
tbc1d24	TBC1 Domain Family Member 24	A1A5K6	4	0
ap2a2	Adaptor Related Protein Complex 2 Subunit Alpha 2	Q5BJ37	2	0
arfgap2	ADP Ribosylation Factor GTPase Activating Protein 2	Q68FK6	2	0
sec23b	SEC23-Like Protein B	Q6DJE0	2	0
sh3bp4-a	SH3 Domain Binding Protein 4	Q6NU22	2	0
**Metalloproteases**				
rnpep	Arginyl Aminopeptidase	Q641C7	2	0
XPNPEP2	Xaa-Pro Aminopeptidase 2	Q52L38	1	0
**Ubiquitination / de-Ubiquitination related factors**				
DTX3	Deltex E3 Ubiquitin Ligase 3	Q4VBG3	2	0
dtl-b	Denticleless protein homolog B	Q6GPU3	2	0
usp5	Ubiquitin Specific Peptidase 5	A0AUT2	2	0
psmf1	Proteasome Inhibitor Subunit 1	Q5XGW2	1	0
znf207	Zinc Finger Protein 207	Q7ZXV8	1	0
asb1	Ankyrin Repeat And SOCS Box Containing 1	Q3KQ26	1	0
ubap2	Ubiquitin Associated Protein 2	Q32N72	1	0
fbxo38	F-Box Protein 38	Q2TAQ0	1	0
n4bp1	NEDD4 Binding Protein 1	Q7ZXG4		
**Etc**				
efnb2	ephrinB2 (self-pulldown)	Q6PF53	100	0
efnb1	ephrinB1	O13097	5	0
katnal2	Katanin p60 ATPase-containing subunit A-like 2	Q3B8D5	2	0

Figure S1. Screening of the novel ephrinB2 binding proteins using mass spectrometric analysis. (A) RNAs of ephrinB2-HA (400pg) were injected into one-cell stage embryos. Co-immunoprecipitation (Co-IP) and mass spectrometric analysis were performed at stage 13. (B) The table indicates a number of possible ephrinB2 binding proteins in the indicated categories.

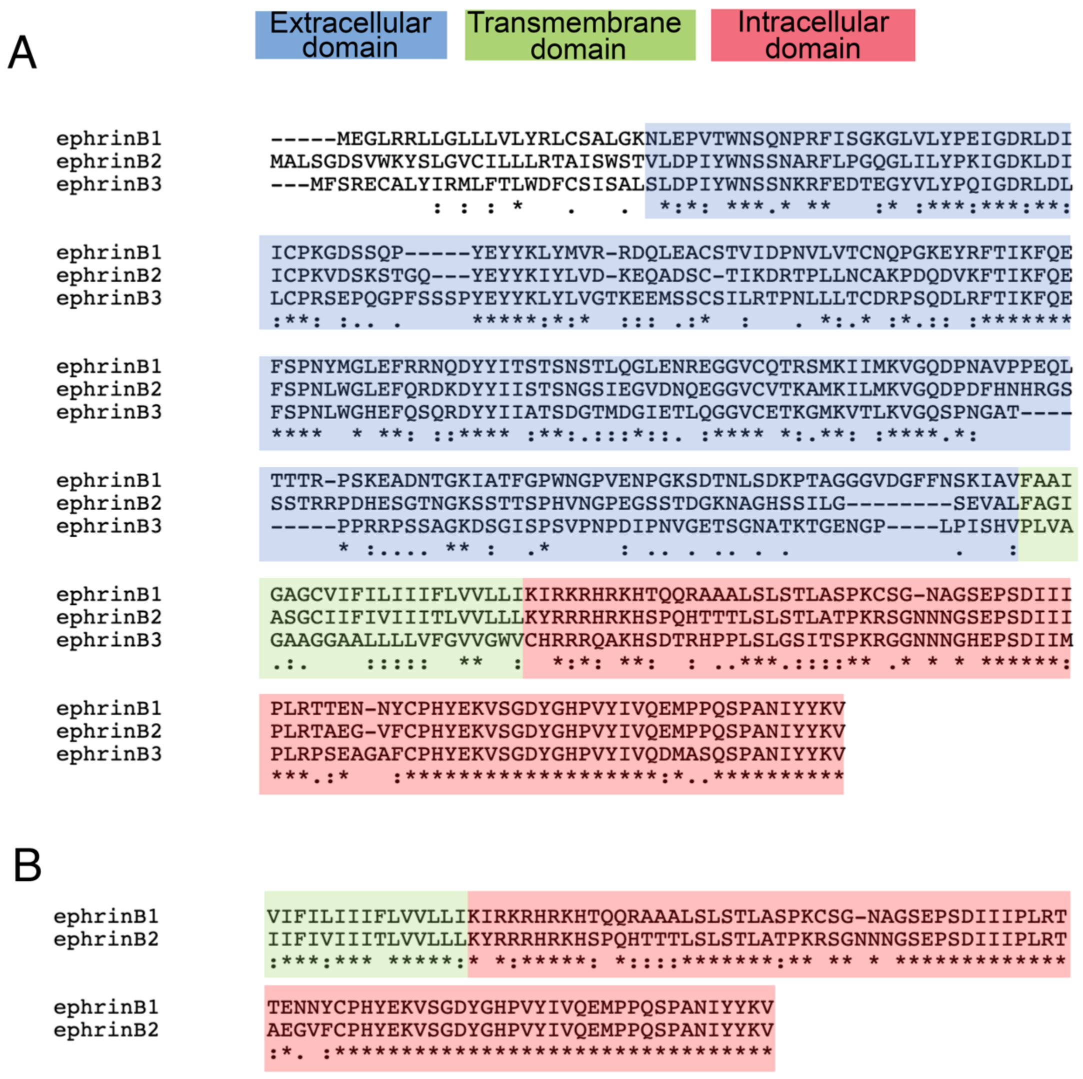

Figure S2. Protein sequence alignment of ephrinBs. (A) Sequence alignment of three ephrinB subfamily members. (B) Sequence alignment of intracellular domain of ephrinB1 and ephrinB2 in pink.

Development • Supplementary information

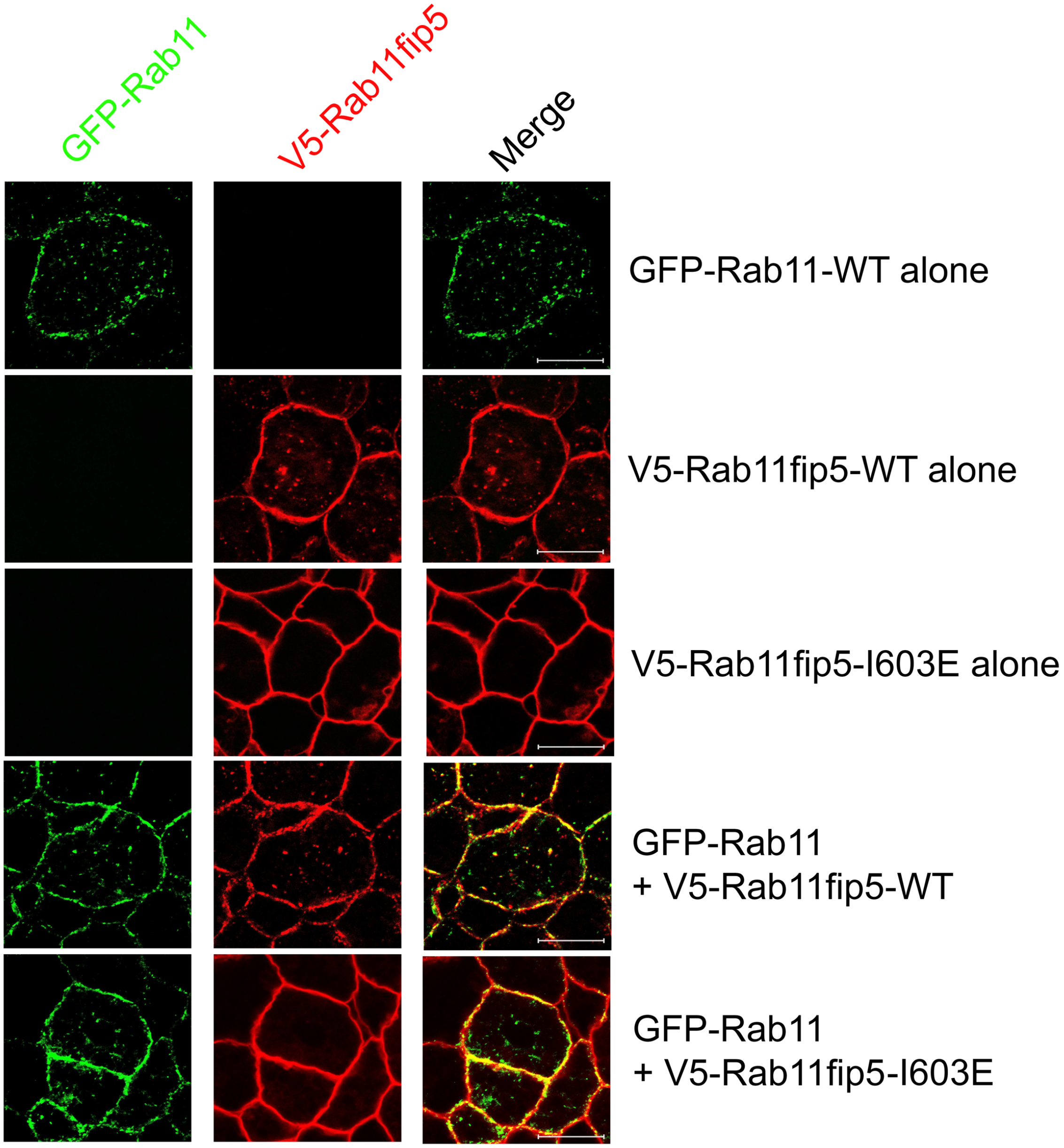

Figure S3. Co-localization of Rab11 and Rab11fip5 is dependent on their interaction. Immuno-staining was performed with ectodermal explants dissected from stage 8 embryos injected with GFP-Rab11 (250pg), V5-Rab11fip5-WT (1ng), and V5-Rab11fip5-I603E (1ng) as indicated. Scale bar, 10μm.

Development • Supplementary information

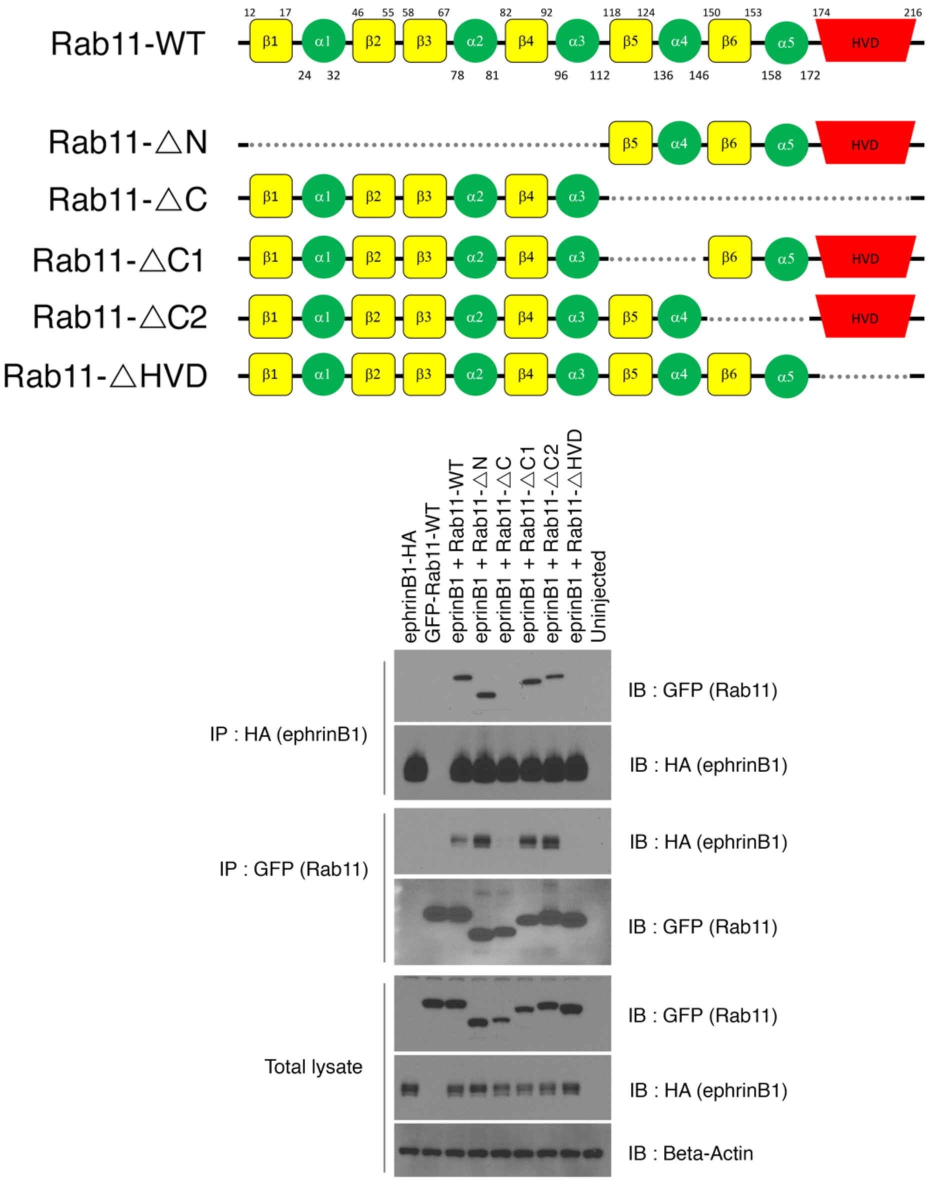

Figure S4. Hypervariable domain (HVD) in Rab11 is required for an interaction with ephrinB1. schematic representation of the domain structure of Rab11. Co-immunoprecipitation assay (Co-IP) was performed using embryos (stage 11) injected with ephrinB1-HA (300pg) and Rab11 deletion mutants (1ng each) as indicated. α: α-helix, β: β-sheet

Development • Supplementary information

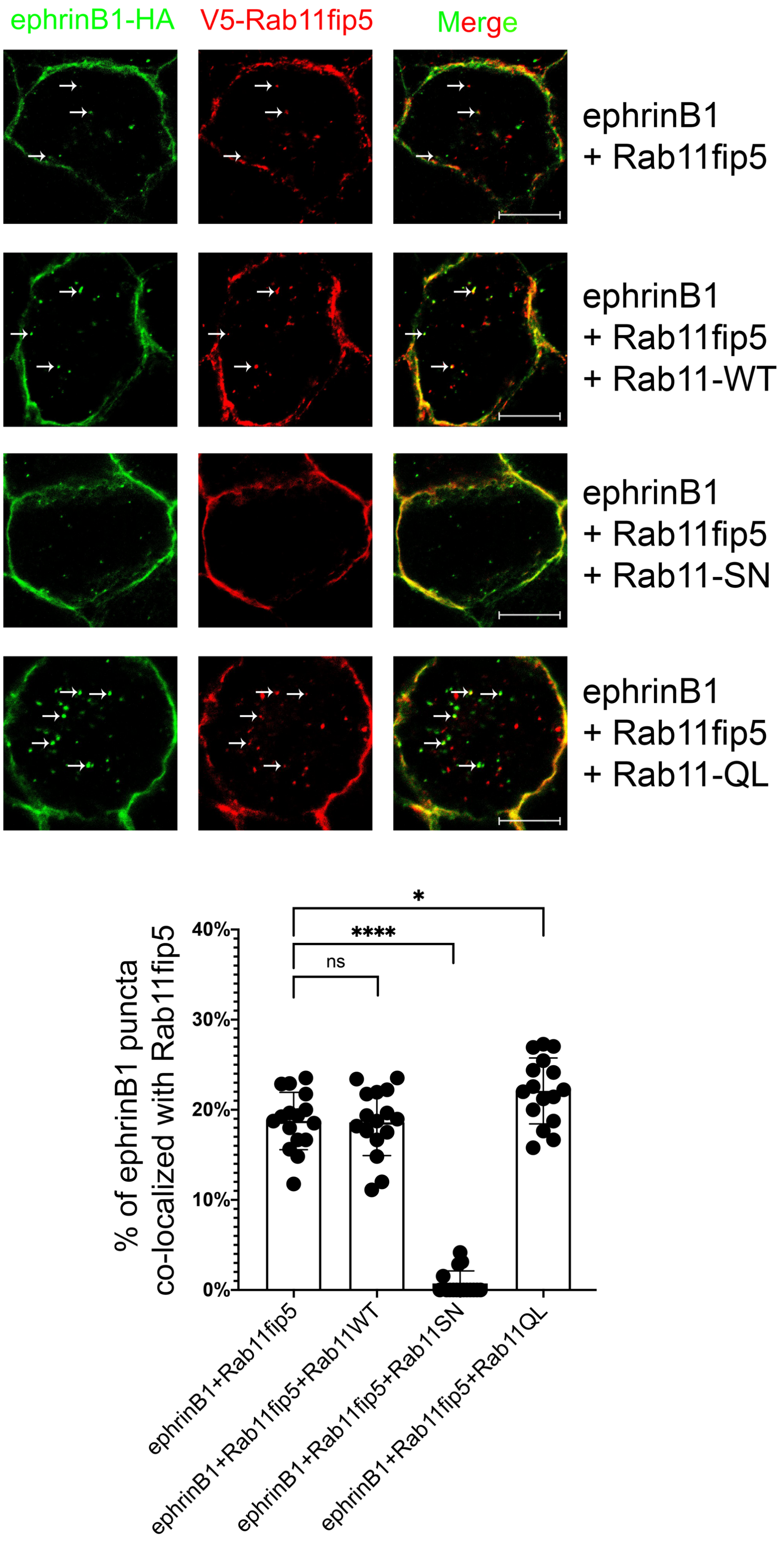

Development • Supplementary information

Figure S5. Rab11 activity affects co-localization of Rab11 and Rab11fip5. Immuno-staining was performed with ectodermal explants dissected from stage 8 embryos injected with ephrinB1-HA (300pg), V5-Rab11fip5-WT (1ng), and Rab11-WT, SN, or QL (1ng) as indicated. White arrows denote the colocalization of Rab11 and Rab11fip5-WT. Scale bar, 10μm. Histogram depicts the percentage of ephrinB1 puncta co-localized with Rab11fip5 in the ectodermal cells (n=15). Quantification with one-way ANOVA (Dunnett's multiple comparisons test), ****$p<0.0001$. Data represent the mean ± S.D. of three individual experiments. * $p < 0.05$, **** $p < 0.0001$, ns: no statistical differences between the groups.

A

ACCCCCCCTAGAAGTTGATGTGGGGCCACTTTCCTTGGACTGAGTTGTGACCCCTAGAGGCTCGTCCT
CATGTTTCTTCGGCCAGAGGCAGAATTGGGCTGGCTCCCTACCCATGTTCAGGTGACTATATTGCAGGC
ACGGGGACTCCGAGCCAAGGGCAAACATGGCACCAGTGATGCGTACACCTTGATCCAGATAGCCCGG
GAGAAGTACAGTACGTCCGTGGTAGAGAAGAGCCCGGGCTCCCCAGAATGGAAGGAGGAATGTTCTT
TCGAACTGGCCCCTGGGGCCTTGGAGCGGGCAGAGAACTGCGACCTTCAGCTGACTGTCATGCACC
GTGCCCTCATTGGTATGGACCAGTTCCTGGGGCAGGTCTCCATCTCACTGCAGCACGTCTTTCAGGAA
GGTCGCTCCCAGAGGAACCAGTGAGTACTACCACATAGCAACCCTTGTCTTCTTG

B

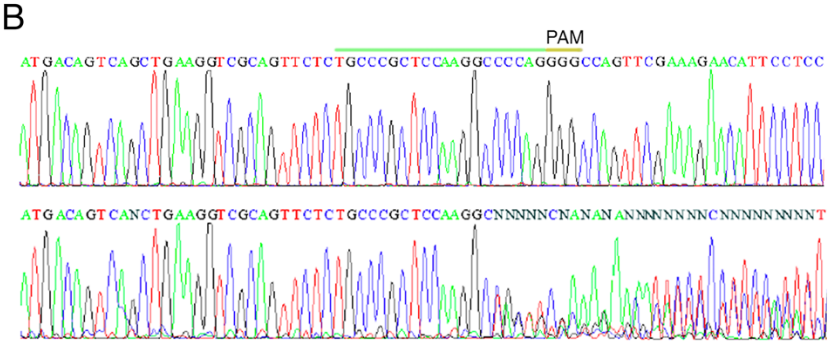

Cas9 alone

Rab11fip5 sgRNA +Cas9

Figure S6. Rab11fip5 knockout using CRSPR/Cas9. Genomic sequences of the first exon of the Rab11fip5 gene. The direct sequencing of PCR amplicons (DSP) shows that several nucleotides were removed in the Cas9 and Rab11fip5 sgRNA injected F0 embryos. A start codon (red), PAM sequences (yellow), Rab11fip5 sgRNA target sequences (green) and gray highlights indicate 5`UTR and intron.

Development • Supplementary information

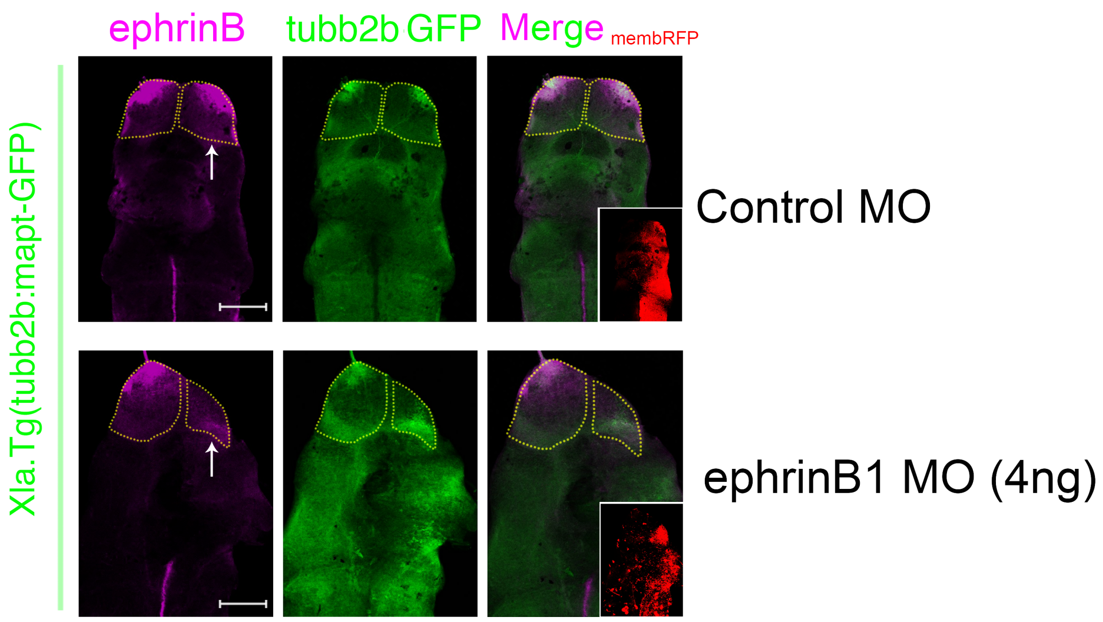

Figure S7. Immunostaining with ephrinB antibody. MOs and membrane-RFP were injected into one D1.1 blastomere at the 16-cell stage as indicated. Brains were dissected at stage 45 and immunostaining performed using anti-ephrinB antibody. The white arrows indicate injected side. Scale bar, 200μm.

Development • Supplementary information

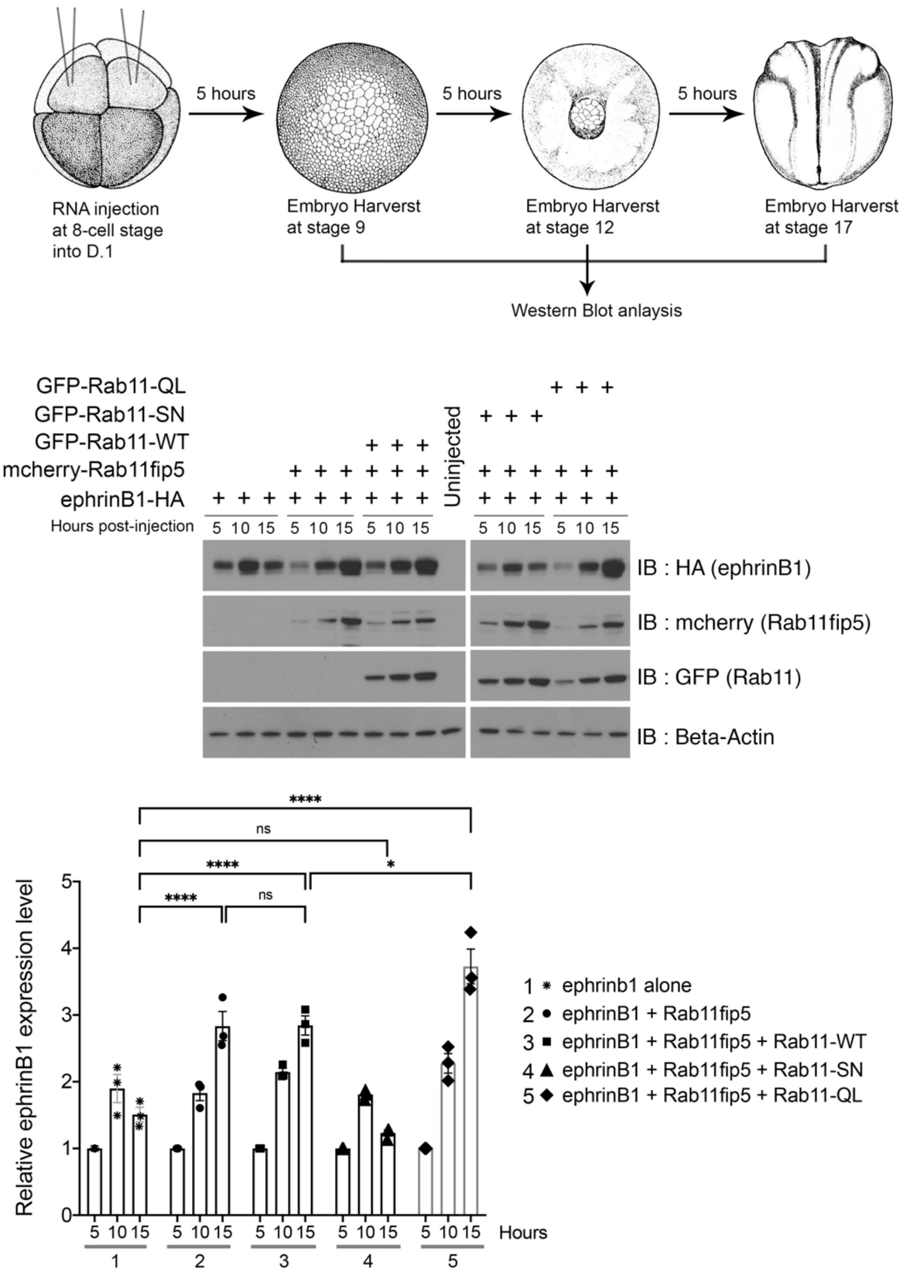

Figure S8. Rab11 activity affects exogenously expressed ephrinB1 levels via Rab11fip5. Schematic representation of experimental procedure. RNAs were injected into two D1 blastomeres at the 8-cell stage as indicated. Embryos were harvested at 5, 10, 15 hours after injection and Western blot analysis was performed. Histogram depicts relative ephrinB1 levels at 15 hours post-injection (n=4). Quantification with one-way ANOVA (Dunnett's multiple comparisons test), ****$p<0.0001$. Data represent the mean ± S.D. of three individual experiments. * $p < 0.05$, **** $p < 0.0001$, ns: no statistical differences between the groups.

Development • Supplementary information

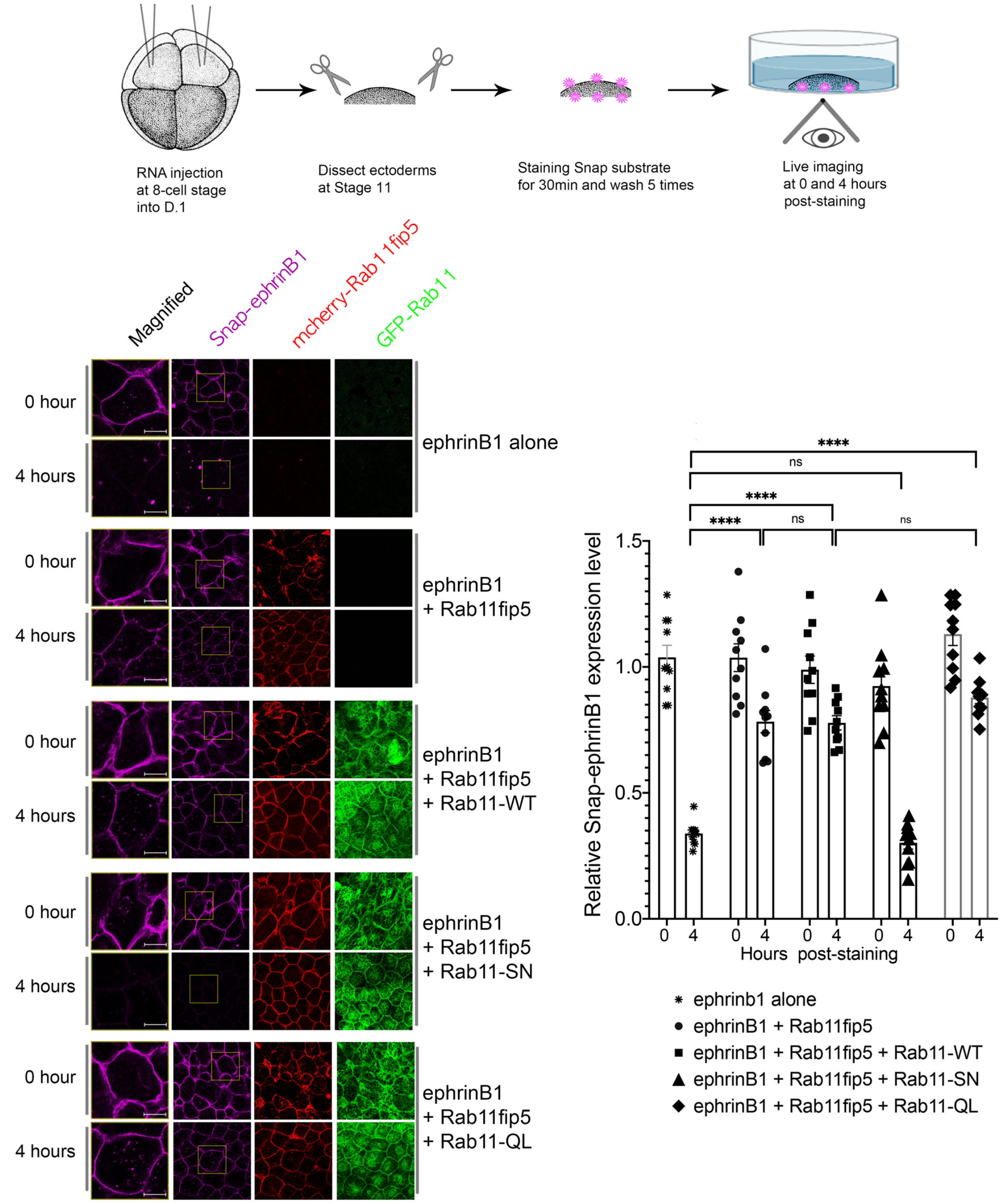

Figure S9. Rab11 activity affects ephrinB1 recycling through Rab11fip5. Schematic representation of experimental procedure. RNAs were injected into two D1 blastomeres at the 8-cell stage as indicated. Ectodermal explants were dissected at stage 11 and then membrane-ephrinB1 was stained using Snap-tag substrate (Non cell permeable, Snap-Surface™ 647). Live cell images were taken at 0 hour and 4hours post-Snap-staining. Scale bar, 10μm. Histogram depicts relative ephrinB1 levels at 0 and 4hours post-injection (n=10). Quantification with one-way ANOVA (Dunnett's multiple comparisons test), ****p<0.0001. Data represent the mean ± S.D. of three individual experiments. **** p < 0.0001, ns: no statistical differences between the groups.

Development • Supplementary information

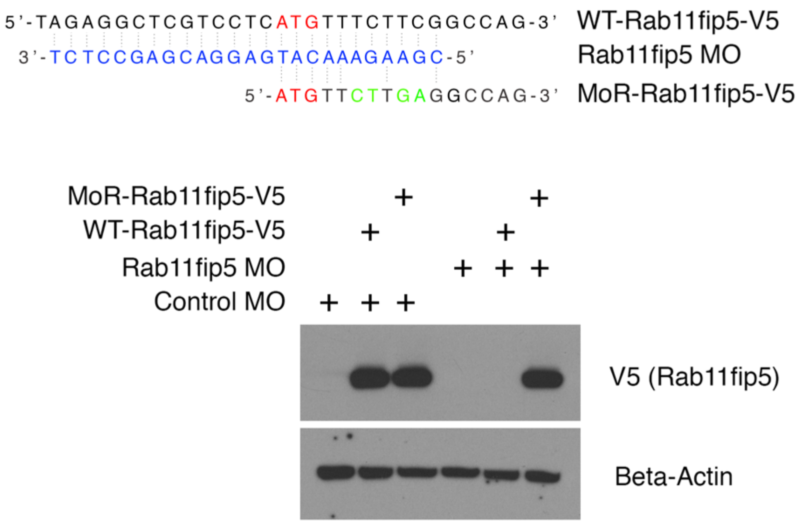

Figure S10. Rab11fip5 morpholino oligos. Rab11fip5 MO targets the translational start region as indicated. Western blot analysis shows that Rab11fip5 MO efficiently blocks exogenous WT-Rab11fip5 expression whereas the expression of the morpholino resistant MoR-Rab11fip5 RNA is not affected by Rab11fip5 MO.

Development • Supplementary information

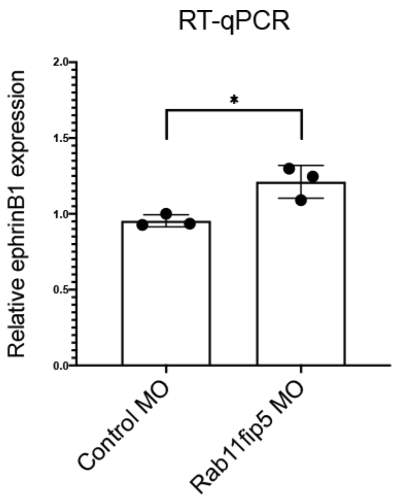

Figure S11. Knockdown of Rab11fip5 did not reduce ephrinB1 mRNA expression. Rab11fip5 MOs were injected into two D1 blastomeres at the 8-cell stage as indicated. Brains were dissected at stage 45 and RT-qPCR analysis was performed. Quantification with unpaired t test, two-tailed (n=3). Data represent the mean ± S.D. of three individual experiments. * $p = 0.0181$.

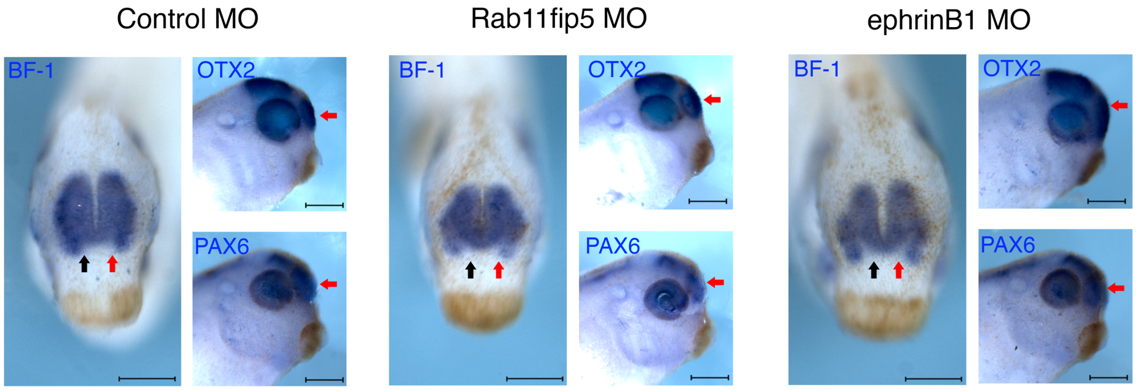

Figure S12. Knockdown of Rab11fip5 did not affect early brain specification. MOs and membrane-RFP were injected into one D1.1 blastomere at the 16-cell stage as indicated. Expression of early brain specification genes were analyzed using WISH at stage 30 with probes for BF-1, OTX2, or PAX6. The black arrows indicate uninjected control side and the red arrows indicate injected side. Scale bar, 200μm.

Development • Supplementary information

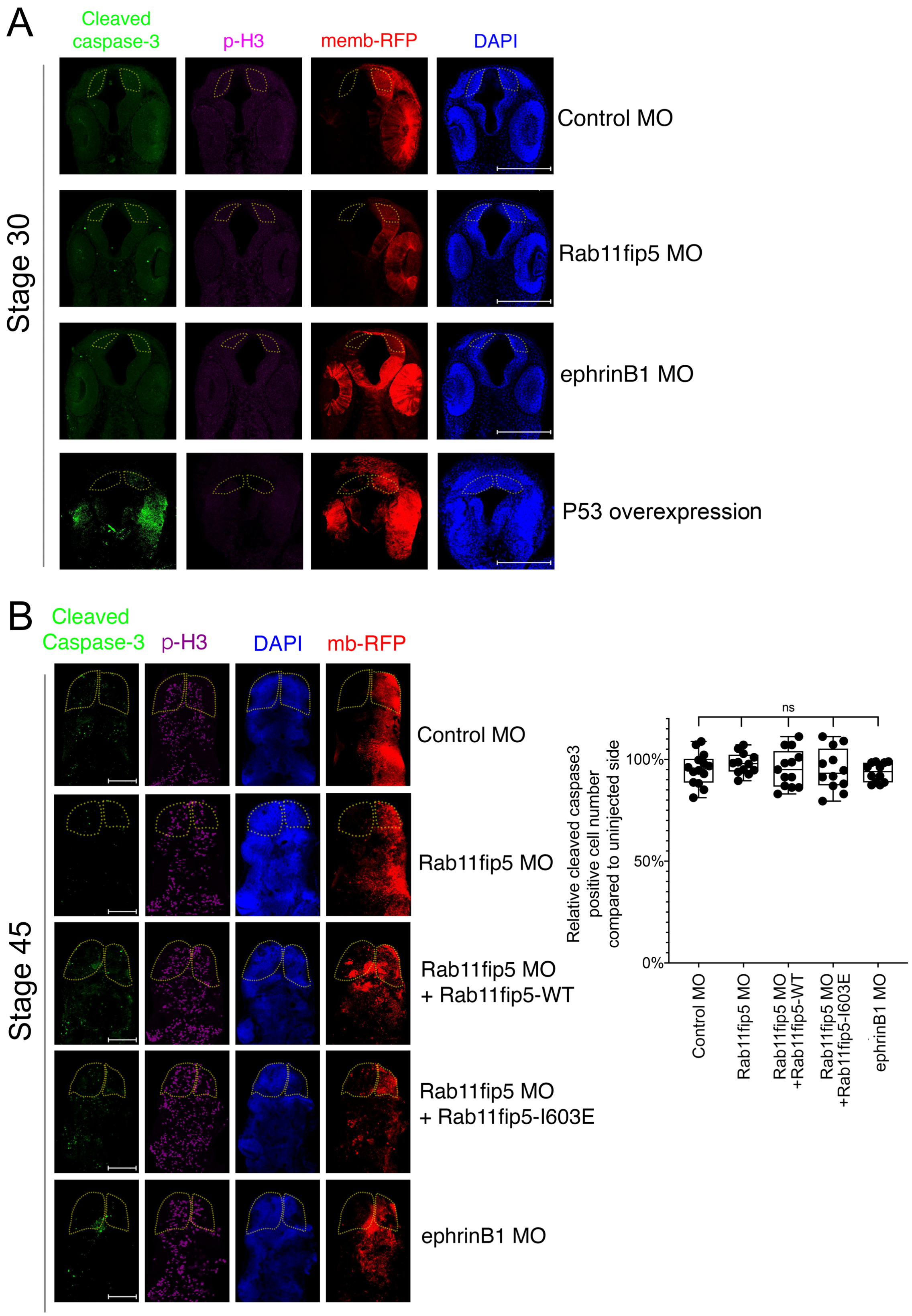

Development • Supplementary information

Figure S13. Knockdown of Rab11fip5 did not induce apoptosis in developing telencephalon. MOs (4ng) along with membrane-RFP (100pg) or p53 RNAs (50pg) were injected into one D1 blastomere at the 8-cell stage as indicated. Brains were dissected at stage 30 (A) or stage 45 (B) and immunostaining performed using anti-Cleaved caspase-3 and phospho-Histone H3 antibodies. Overexpression of p53 is used as a positive control. Scale bar, 200μm. Histograms depict relative Cleaved caspase-3 positive cell numbers compared with uninjected telencephalons (n=12). Quantification with one-way ANOVA (Dunnett's multiple comparisons test), p = 0.7218. Data represent the mean ± S.D. of three individual experiments. ns: no statistical differences between the groups.
