# Supplementary material for: Rab11fip5 regulates telencephalon development via ephrinB1 recycling
Source: Development. 2021 Feb 2;148(3):dev196527. doi: 10.1242/dev.196527 (PMC7875491; doi:10.1242/dev.196527)
Supplement: Reviewer comments [file develop-148-196527_review_history.pdf]

Rab11fip5 regulates telencephalon development via ephrinB1 recycling.

Jaeho Yoon, Jerlin Garo, Moonsup Lee, Jian Sun, Yoo-Seok Hwang and Ira O. Daar
DOI: 10.1242/dev.196527

Editor: François Guillemot

Review timeline

Original submission:	1 September 2020
Editorial decision:	8 October 2020
First revision received:	22 December 2020
Accepted:	25 December 2020

Original submission

First decision letter

MS ID#: DEVELOP/2020/196527

MS TITLE: Rab11fip5 regulates telencephalon development via ephrinB1 recycling

AUTHORS: Jaeho Yoon, Jerlin Garo, Moonsup Lee, Jian Sun, Yoo-Seok Hwang, and Ira O Daar

I have now received the reports of three referees on your manuscript and I have reached a decision. The referees' comments are appended below, or you can access them online: please go to BenchPress and click on the 'Manuscripts with Decisions' queue in the Author Area.

As you will see, the referees express considerable interest in your work, but two of them also have significant criticisms and recommend a substantial revision of your manuscript before we can consider publication. In particular, referee 2 suggests that you determine the half-life of the ephrin B1 protein. If you are able to revise the manuscript along the lines suggested, which may involve further experiments, I will be happy to receive a revised version of the manuscript. Determining how the inactivation of ephrinB1 affects cell proliferation in the telencephalon, a question raised by referee 1, seems indeed beyond the scope of the paper and is not required for you to submit your revised manuscript. The revised paper will be re-reviewed by the original referees, and its acceptance will depend on your addressing satisfactorily all their major concerns. Please also note that Development will normally permit only one round of major revision.

We are aware that you may be experiencing disruption to the normal running of your lab that make experimental revisions challenging. If it would be helpful, we encourage you to contact us to discuss your revision in greater detail. Please send us a point-by-point response indicating where you are able to address concerns raised (either experimentally or by changes to the text) and where you will not be able to do so within the normal timeframe of a revision. We will then provide further guidance. Please also note that we are happy to extend revision timeframes as necessary.

Please attend to all of the reviewers' comments and ensure that you clearly highlight all changes made in the revised manuscript. Please avoid using 'Tracked changes' in Word files as these are lost in PDF conversion. I should be grateful if you would also provide a point-by-point response detailing how you have dealt with the points raised by the reviewers in the 'Response to Reviewers' box. If you do not agree with any of their criticisms or suggestions please explain clearly why this is so.

© 2021. Published by The Company of Biologists under the terms of the Creative Commons Attribution License (https://creativecommons.org/licenses/by/4.0/).

Reviewer 1

Advance summary and potential significance to field

This manuscript investigates the cellular and molecular mechanism by which ephrin endocytosis is regulated in the telencephalon of amphibian embryos. They identify Rab11fip5 as candidate for this regulation. They show that Rab11fip5 and EphrinB1 are co-expressed in the telencephalon, and co-immunoprecipitation analysis shows that they physically interact. Inhibition of Rab11fip5 or EphrinB1leads to a reduction in telencephalon size, and inhibition of Rab11fip5 decreases the levels of EphrinB1 in the telencephalon. The authors show that Rab11fip5 regulates the recycling of EphrinB1, showing that Rab11fip5 inhibition leads to depletion of EphrinB1 in the cell membrane. To explain the reduced size of the telencephalon they show that Rab11fip5 depletion inhibits cell proliferation. Importantly, Rab11fip5 depletion phenotypes (telencephalon size and cell proliferation) can be rescued by expressing EphrinB1, supporting the notion that EphrinB1 is the Rab11fip5 target.

Comments for the author

This work represents an important contribution to our understanding of Ephrin recycling. Ephrins play a multitude of function in different systems and tissues, and therefore, understanding the molecular regulation of this family of protein will be of wide interest for the cell and developmental biology communities at large.
The experiments are clear and convincing and I have mainly minor comments.

My major problem with this paper is that while the cell biology experiments are nicely done, the developmental biology questions is not properly addressed. In other words, there is no clear analyse of the phenotype from a developmental perspective. The phenotypes that they describe is a reduction of telencephalon size when recycling of EphrinB1 is impaired. But how is this phenotypes generated remains unexplored. The authors show an effect on cell proliferation, but how Ephrinb1 recycling affect cell proliferation? The authors also mention that “ephrinB1 acts to prevent the differentiation of progenitor cells and maintain them in a proliferative state”; but this is never tested. Did the authors observe an increased in progenitors of the telencephalon? I believe that these are important questions for a journal like Development but at the same time I would understand if the authors argue that these questions go beyond the scope of the article.

Minor comment:

In Fig 4B the authors show that the amount of ephrinB1 was reduced by approximately 40%. It is not clear how this % was calculated. As the size of the telencephalon is also reduced in the experimental side, they should normalize the levels of ephrinB1 expression by the area of the telencephalon. In addition, the levels of ephrinB1 mRNA should also be analysed, to support the author claim that ephrin levels are modulated at the protein and not the mRNA level.

Reviewer 2

Advance summary and potential significance to field

This paper brings forth a novel player in Eph/ephrin signaling. Rab11fip5 interacts with ephrinB1 through Rab11, and that interaction affects telencephalon development in the Xenopus embryo. The authors pin down the critical domains and conditions for proper functioning of the complex. For instance, Rab11 can only complete the interaction when it is in its active state and binds to the proper residue of the RBD domain of Rab11fip5, and they in turn bind to the PDZ binding motif of ephrinB1.
In the developing Xenopus embryo, inhibition of Rab11fip5 or ephrinB1 leads to the reduction in size and proliferation rate of the telencephalon, which can be rescued by WT Rab11fip5 or ephrinB1. As a conclusion, the authors suggest that the Rab11/Rab11fip5 complex takes ephrinB1 as cargo to regulate its proper recycling.

© 2021. Published by The Company of Biologists under the terms of the Creative Commons Attribution License (https://creativecommons.org/licenses/by/4.0/).

Overall, the authors present a careful examination of the interaction between ephrinB1 and Rab11fip5 and its meaning in telencephalon development. They shed light on an important regulatory mechanism of Eph/ephrin signaling.
This paper is interesting, and its hypotheses are accompanied with compelling data. I would, however, suggest a few changes and additions to improve the quality and eliminate confusion.
JKH

Comments for the author

1. The text associated with figure 2 claims that because mutated Rab11fip5 fails to interact with both ephrinB1 and Rab11, Rab11 must be the link between Rab11fip5 and ephrinB1. This deduction is missing a major link, which is the interaction between ephrinB1 and Rab11. Surprisingly, this piece of the puzzle comes in the following paragraph. I suggest moving figure 3b to figure 2, as it will eliminate confusion.
2. Similarly, paragraph 3 of the results contains a concluding sentence
(ephrinB1 is cargo of the Rab11 complex) that reads more like unsubstantiated foreshadowing than a summary of the presented results.
3. Constitutively active Rab11 was used in only one figure, to confirm its interaction with ephrinB1. Figure 3d and figure 5 are in need of this construct.
In both cases, inhibition by inactive Rab11 was interpreted as induction by Rab11. While the correlation is understandable, I believe using QL Rab11 would clarify and strengthen the evidence.
4. If possible, the interaction domains of Rab11 could be presented, since those of ephrinB1 and Rab11fip5 were discussed extensively.
5. Figure 7 and its associated paragraph in the results section discusses the short half-life of ephrinB1 RNA. The issue is remedied with the use of DNA instead. Then, the low level of tagged ephrinB1 is interpreted as a result of recycling. While that may indeed be the case, one can suspect that the injected plasmids also face a short lifespan. I suggest an experiment quantifying ephrinB1 half-life under various conditions to eliminate doubt.
6. While the results section includes strong claims and conclusions, the discussion section appears timid and lacks a confident discussion of the significance of the obtained results. I suggest further elaboration on the claims at the end of each paragraph of the results section, as well as a discussion of the limitations and future directions of this study.

Minor issues
A. Figure 3a mentions Rip11, which is never identified anywhere in the paper.
B. While unnecessary for this study, it will be interesting to perform a mass spectrometry analysis on ephrinB1 to see its specific regulators and interacting partners.
C. Typos:
- page 5: ephinB2-HA
- page 6: to examined
- page 13: laevies
- page 14: dehydrate

<u>Reviewer 3</u>

Advance summary and potential significance to field

With apologies for the delayed review, I congratulate the authors for an interesting and well-documented submission. As succinctly presented in the abstract, the authors have shown a requirement for recycling of EphrinB. This is shown by exploiting Rab11fip5, and Rab11 and EphrinB interacting protein. By mutagenizing the Rab11fip5 domain that interacts with Rab11, it is clearly shown that the intermediate Rab11fip5 is needed to mediate the Ephrinb/Rab11 interaction, such that the complete complex is needed to maintain the stability of Ephrinb on the membrane through its recycling.
In the phenotypic characterization, it is also shown that the function of the complex is required to maintain proper proliferation and forebrain size in tadpoles. This is done with a variety of excellent expression of mutants, and morpholino knockdown, where both Rab11fip5 wild type function and proper expression of EphrinB are needed. Indeed, overexpression of Ephrin B can rescue the

© 2021. Published by The Company of Biologists under the terms of the Creative Commons Attribution License (https://creativecommons.org/licenses/by/4.0/).

phenotype. This paper therefore shows this important role for recycling, and also addresses the potential role for a defective complex in causing forebrain proliferation defects, and autism in humans.
My only question is whether the transgenic Tubb promoter and 5Â'UTR is used rather than the stated 5Â'UTP

Comments for the author

Accept as is.

First revision

Author response to reviewers' comments

We are submitting a revised manuscript (ID#: DEVELOP/2020/196527) that is entitled "**Rab11fip5 regulates telencephalon development via ephrinB1 recycling**". We greatly appreciate the suggestions and comments of the editor and reviewers, and a number of new experiments have been performed to address the concerns. We feel that in this revised paper, our use of a combination of loss- of-function (endogenous) experiments, replacement experiments (knockdown of endogenous protein and re-expression at carefully titrated levels), and biochemical assays in *Xenopus* embryos provide mechanistic insight into how Rab11fip5 plays a role in regulating telencephalon development via recycling of the critical cargo ephrinB1. We are grateful to the reviewers whose suggestions have led to a more thorough assessment of Rab11fip5 and its role in this process, and to strengthen the claims of our paper.

The concerns of reviewers and editor have been addressed below:

Reviewer 1 Advance Summary and Potential Significance to Field:
This manuscript investigates the cellular and molecular mechanism by which ephrin endocytosis is regulated in the telencephalon of amphibian embryos. They identify Rab11fip5 as candidate for this regulation. They show that Rab11fip5 and EphrinB1 are co-expressed in the telencephalon, and co-immunoprecipitation analysis shows that they physically interact. Inhibition of Rab11fip5 or EphrinB1leads to a reduction in telencephalon size, and inhibition of Rab11fip5 decreases the levels of EphrinB1 in the telencephalon. The authors show that Rab11fip5 regulates the recycling of EphrinB1, showing that Rab11fip5 inhibition leads to depletion of EphrinB1 in the cell membrane. To explain the reduced size of the telencephalon they show that Rab11fip5 depletion inhibits cell proliferation. Importantly, Rab11fip5 depletion phenotypes (telencephalon size and cell proliferation) can be rescued by expressing EphrinB1, supporting the notion that EphrinB1 is the Rab11fip5 target.

Reviewer 1 Comments for the Author:
This work represents an important contribution to our understanding of Ephrin recycling. Ephrins play a multitude of function in different systems and tissues, and therefore, understanding the molecular regulation of this family of protein will be of wide interest for the cell and developmental biology communities at large. The experiments are clear and convincing and I have mainly minor comments.

We appreciate the reviewer's overall assessment of the manuscript.

My major problem with this paper is that while the cell biology experiments are nicely done, the developmental biology questions is not properly addressed. In other words, there is no clear analyse of the phenotype from a developmental perspective. The phenotypes that they describe is a reduction of telencephalon size when recycling of EphrinB1 is impaired. But how is this phenotypes generated remains unexplored. The authors show an effect on cell proliferation, but how Ephrinb1 recycling affect cell proliferation? The authors also mention that "ephrinB1 acts to prevent the differentiation of progenitor cells and maintain them in a proliferative state"; but this is never

© 2021. Published by The Company of Biologists under the terms of the Creative Commons Attribution License (https://creativecommons.org/licenses/by/4.0/).

tested. Did the authors observe an increased in progenitors of the telencephalon? I believe that these are important questions for a journal like Development, but at the same time I would understand if the authors argue that these questions go beyond the scope of the article.

We agree with the reviewer that these are fascinating questions. While we mention that the EphA4 receptor affects proliferation in cortical progenitors in mice, the mechanism has not been addressed by anyone in the Eph/ephrin field. We believe such an undertaking is beyond the scope of our current study.

We also agree with the reviewer that the differentiation and maintenance of progenitor cells would be excellent for future studies to provide significant mechanistic insight and knowledge of these signaling pathways. However, we believe our results in the current study provide a novel mechanistic connection between the candidate autism spectrum disorder gene product, Rab11fip5, and ephrinB1, and indicates that proper recycling of ephrinB1 through the Rab11/Rab11fip5 complex controls proper telencephalon formation.

Although it does not directly address the reviewer's comment, we should mention that we have also performed whole mount in situ hybridization with early brain markers and an apoptosis marker to strength our conclusion that Rab11fip5 regulates cell proliferation in the developing telencephalon through control of ephrinB1 levels (Fig S12 and S13).

<u>Minor comment:</u>

In Fig 4B the authors show that the amount of ephrinB1 was reduced by approximately 40%. It is not clear how this % was calculated. As the size of the telencephalon is also reduced in the experimental side, they should normalize the levels of ephrinB1 expression by the area of the telencephalon.

We agree with the reviewer that we were not clear about our calculation. The results are now displayed and noted as a comparison of ephrinB1 levels between the injected and uninjected side within the embryo. We feel this is the most clear and direct way to assess the change in ephrinB1 levels. We did not display the data as suggested by the reviewer (normalizing to the area of the telencephalon) since the reduction in area is caused by the decrease in ephrinB1 levels. Thus, the telencephalic area will be reduced to some degree in proportion to the ephrinB1 decrease. Below for the reviewer is a display of both sets of calculations:

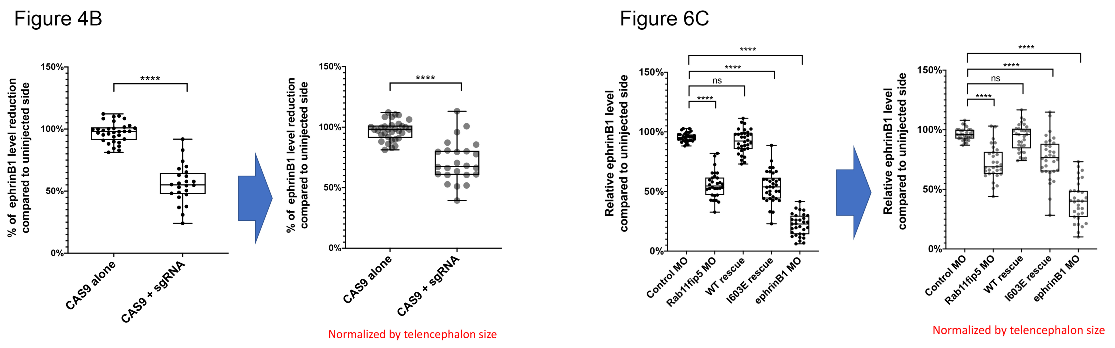

While we note that the trends are quite similar whether we compare ephrin B1 levels between the injected to uninjected side or whether we normalize ephrinB1 levels to area first, we prefer the direct comparison. However, if the reviewer feels strongly about this issue, we can display the normalized comparison.

In addition, the levels of ephrinB1 mRNA should also be analysed, to support the author claim that ephrin levels are modulated at the protein and not the mRNA level.

We thank the reviewer for this suggestion and excellent point. We have now analyzed the levels of ephrinB1 mRNA by qPCR in embryonic brains of control and Rab11fip5 morphants (Fig S11). We actually find a very modest but reproducible increase in ephrinB1 mRNA in the Rab11fip5 morphants;

© 2021. Published by The Company of Biologists under the terms of the Creative Commons Attribution License (https://creativecommons.org/licenses/by/4.0/).

indicating that loss of ephrinB1 is a post-translational event.

Reviewer 2 Advance Summary and Potential Significance to Field:

This paper brings forth a novel player in Eph/ephrin signaling. Rab11fip5 interacts with ephrinB1 through Rab11, and that interaction affects telencephalon development in the Xenopus embryo. The authors pin down the critical domains and conditions for proper functioning of the complex. For instance, Rab11 can only complete the interaction when it is in its active state and binds to the proper residue of the RBD domain of Rab11fip5, and they in turn bind to the PDZ binding motif of ephrinB1. In the developing Xenopus embryo, inhibition of Rab11fip5 or ephrinB1 leads to the reduction in size and proliferation rate of the telencephalon, which can be rescued by WT Rab11fip5 or ephrinB1. As a conclusion, the authors suggest that the Rab11/Rab11fip5 complex takes ephrinB1 as cargo to regulate its proper recycling.

Overall, the authors present a careful examination of the interaction between ephrinB1 and Rab11fip5 and its meaning in telencephalon development. They shed light on an important regulatory mechanism of Eph/ephrin signaling. This paper is interesting, and its hypotheses are accompanied with compelling data. I would, however, suggest a few changes and additions to improve the quality and eliminate confusion.

We thank the reviewer for the assessment of our manuscript as being complete and of high quality.

Reviewer 2 Comments for the Author:

1. The text associated with figure 2 claims that because mutated Rab11fip5 fails to interact with both ephrinB1 and Rab11, Rab11 must be the link between Rab11fip5 and ephrinB1. This deduction is missing a major link, which is the interaction between ephrinB1 and Rab11. Surprisingly, this piece of the puzzle comes in the following paragraph. I suggest moving figure 3b to figure 2, as it will eliminate confusion.

The reviewer makes an excellent point and we have now changed the order of the figure panels and text to make a more logical flow regarding the Rab11fip5/Rab11/ephrinB1 interaction.

2. Similarly, paragraph 3 of the results contains a concluding sentence (ephrinB1 is cargo of the Rab11 complex) that reads more like unsubstantiated foreshadowing than a summary of the presented results.

As suggested, we have revised the text.

3. Constitutively active Rab11 was used in only one figure, to confirm its interaction with ephrinB1. Figure 3d and figure 5 are in need of this construct. In both cases, inhibition by inactive Rab11 was interpreted as induction by Rab11. While the correlation is understandable, I believe using QL Rab11 would clarify and strengthen the evidence.

We have now included experiments with Rab11-QL and are now presented in Fig S5, S8, and S9 to strengthen the role of Rab11 in the process.

4. If possible, the interaction domains of Rab11 could be presented, since those of ephrinB1 and Rab11fip5 were discussed extensively.

The reviewer makes an excellent point. We have now determined the interaction motif of Rab11. Our result showed that Hypervariable domain (HVD) in C-terminal region of Rab11 is required for an interaction with ephrinB1. This domain is different than the RBD domain which is highly conserved in all

Rab11fip family proteins, that directly interacts with Switch regions in the N-terminal region of Rab11 (Jagoe et al., 2006). This data is now presented in Fig. S4.

5. Figure 7 and its associated paragraph in the results section discusses the short half-life of

© 2021. Published by The Company of Biologists under the terms of the Creative Commons Attribution License (https://creativecommons.org/licenses/by/4.0/).

ephrinB1 RNA. The issue is remedied with the use of DNA instead. Then, the low level of tagged ephrinB1 is interpreted as a result of recycling. While that may indeed be the case, one can suspect that the injected plasmids also face a short lifespan. I suggest an experiment quantifying ephrinB1 half-life under various conditions to eliminate doubt.

We are sorry for the confusion, which is likely due to our brevity when describing a technical hurdle that we overcame by use of DNA constructs containing a neural beta-tubulin (tubb2b) promoter to express an HA-tagged ephrinB1 (NTB-ephrinB1-HA; Fig. 7A) to rescue the Rab11Fip5 knockdown telencephalic phenotype. The reduced telencephalon size in Rab11Fip5 morphant embryos is a late-stage phenotype. Thus, using our standard rescue method of injecting mRNA for ephrinB1 in a 32 cell stage embryo results in undetectable ectopic expressed ephrinB1 protein at the late stages of development. This is most likely due to a combination of ephrinB1 mRNA degradation *in vivo* over time and the relatively short half-life of ephrinB1 protein (about 2.5 hours at the neurula stage that we determined in a previous Genes & Development paper, 2014). One reason this was not solved by injecting more RNA (beyond the low doses we attempted) was that over-expressing ephrinB1 at high levels in early embryogenesis leads to multiple defects that would prevent a clear rescue of the late-stage phenotype. We now more clearly explain the issue and resolution regarding Figure 7.

We have also added another experiment which may also yield some clarification. We have now analyzed the levels of ephrinB1 mRNA by qPCR in embryonic brains of control and Rab11fip5 morphants (Fig S11). We actually find a very modest but reproducible increase in ephrinB1 mRNA in the Rab11fip5 morphants; clearly indicating that loss of ephrinB1 is a post-translational event.

6. While the results section includes strong claims and conclusions, the discussion section appears timid and lacks a confident discussion of the significance of the obtained results. I suggest further elaboration on the claims at the end of each paragraph of the results section, as well as a discussion of the limitations and future directions of this study.

While the reviewer’s point is well taken, we believe that we should make succinct clear statements regarding our concluding statements at the end of each result. However, as suggested by the reviewer, we now expanded our discussion points (bottom page 13).

Minor issues
A. Figure 3a mentions Rip11, which is never identified anywhere in the paper.

It has been corrected.

B. While unnecessary for this study, it will be interesting to perform a mass spectrometry analysis on ephrinB1 to see its specific regulators and interacting partners.

We agree with the reviewer. Several years ago, we performed such an analysis, and it formed the basis of several subsequent reports identifying interactors (Dvl, Stat3, Smurf, flotillin, and CNK1).

C. Typos:
- *page 5: ephinB2-HA*
- *page 6: to examined*
- *page 13: laevies*
- *page 14: dehydrate*

We have made the corrections.

Reviewer 3 Advance Summary and Potential Significance to Field:
With apologies for the delayed review, I congratulate the authors for an interesting and well-documented submission. As succinctly presented in the abstract, the authors have shown a requirement for recycling of EphrinB. This is shown by exploiting Rab11fip5, and Rab11 and EphrinB interacting protein. By mutagenizing the Rab11fip5 domain that interacts with Rab11, it is clearly shown that the intermediate Rab11fip5 is needed to mediate the Ephrinb/Rab11 interaction, such that the complete complex is needed to maintain the stability of Ephrinb on the membrane through its recycling. In the phenotypic characterization, it is also shown that the function of the complex

© 2021. Published by The Company of Biologists under the terms of the Creative Commons Attribution License (https://creativecommons.org/licenses/by/4.0/).

is required to maintain proper proliferation and forebrain size in tadpoles. This is done with a variety of excellent expression of mutants, and morpholino knockdown, where both Rab11fip5 wild type function and proper expression of EphrinB are needed. Indeed, overexpression of Ephrin B can rescue the phenotype. This paper therefore shows this important role for recycling, and also addresses the potential role for a defective complex in causing forebrain proliferation defects, and autism in humans.

We thank the reviewer for his assessment of the study.

My only question is whether the transgenic Tubb promoter and 5'UTR is used rather than the stated 5'UTP.

We made the correction.

Reviewer 3 Comments for the Author:

Accept as is.

Again, we thank the reviewer for his assessment.

We thank the reviewers and the editor for their insightful and thorough comments and suggestions. We have provided additional experiments as requested. We believe that we have addressed the major scientific concerns. We feel that the reviewers' suggestions have significantly strengthened the claims of our paper and we look forward to your positive assessment of this revised manuscript.

Second decision letter

MS ID#: DEVELOP/2020/196527

MS TITLE: Rab11fip5 regulates telencephalon development via ephrinB1 recycling.

AUTHORS: Jaeho Yoon, Jerlin Garo, Moonsup Lee, Jian Sun, Yoo-Seok Hwang, and Ira O Daar
ARTICLE TYPE: Research Article

I am delighted to tell you that your manuscript has been accepted for publication in Development, pending our standard ethics checks.

© 2021. Published by The Company of Biologists under the terms of the Creative Commons Attribution License (https://creativecommons.org/licenses/by/4.0/).
